# Estimating the Under-Five Mortality Rate Using a Bayesian Hierarchical Time Series Model

**DOI:** 10.1371/journal.pone.0023954

**Published:** 2011-09-28

**Authors:** Leontine Alkema, Wei Ling Ann

**Affiliations:** Department of Statistics and Applied Probability, National University of Singapore, Singapore, Singapore; Genentech Inc., United States of America

## Abstract

**Background:**

Millennium Development Goal 4 calls for a reduction in the under-five mortality rate by two-thirds between 1990 and 2015, which corresponds to an annual rate of decline of 4.4%. The United Nations Inter-Agency Group for Child Mortality Estimation estimates under-five mortality in every country to measure progress. For the majority of countries, the estimates within a country are based on the assumption of a piece-wise constant rate of decline.

**Methods and Findings:**

This paper proposes an alternative method to estimate under-five mortality, such that the underlying rate of change is allowed to vary smoothly over time using a time series model. Information about the average rate of decline and changes therein is exchanged between countries using a Bayesian hierarchical model. Cross-validation exercises suggest that the proposed model provides credible bounds for the under-five mortality rate that are reasonably well calibrated during the observation period. The alternative estimates suggest smoother trends in under-five mortality and give new insights into changes in the rate of decline within countries.

**Conclusions:**

The proposed model offers an alternative modeling approach for obtaining estimates of under-five mortality which removes the restriction of a piece-wise linear rate of decline and introduces hierarchy to exchange information between countries. The newly proposed estimates of the rate of decline in under-5 mortality and the uncertainty assessments would help to monitor progress towards Millennium Development Goal 4.

## Introduction

Millennium Development Goal 4 (MDG4) calls for a reduction in the under-five mortality rate by two-thirds between 1990 and 2015. The United Nations Inter-Agency Group for Child Mortality Estimation (IGME) estimates under-five mortality in every country to measure progress. Its estimates are revised every year; the most recent estimates were published in September 2010 [1], [2]. Measurements of the under-five mortality rate (U5MR) are available from different sources, including vital registration systems and sample surveys, and are published by IGME in the data base *CME Info* (www.childmortality.org). A set of standard weights that quantify the relative accuracy of different types of observations has been developed [3], [4]. In this weighting system, observations that are deemed to be of high quality are assigned a higher weight than observations of lesser quality. Observations from sources that are known to be over- or underestimating under-five mortality are assigned a zero weight.

For the majority of countries, IGME uses a linear spline regression estimation approach for estimating U5MR [2]. In short, the observation period is broken up into different periods and a constant rate of decline is estimated within each period. The number of periods and their start and end points depend on the availability of observations and the weight that has been assigned to the observation. The estimated rate of decline within the period is estimated using weighted least-squares estimation.

**Figure 1:**
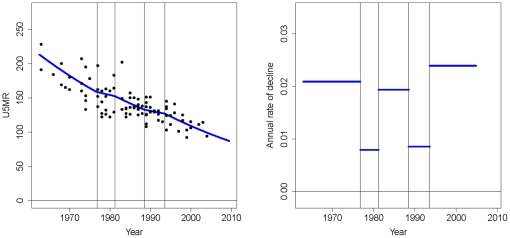
Illustration of the spline estimation approach for Pakistan. Left: U5MR against time. Observations are represented by black dots, spline estimates are represented by the blue line. Right: Underlying annual rate of decline of the spline estimates against time. The vertical lines in both plots represent the time points (knots) at which the rate of decline changes.

The spline estimation approach is illustrated in Figure 1 for Pakistan. The first plot shows the observations of U5MR expressed as the number of deaths per 1,000 live births (black dots) and the spline estimates. The vertical lines represent the time points, which are called the knots, at which the rate of decline is allowed to change. The second plot shows the annual rate of decline, given by log(U5MR(t-1)/U5MR(t)), which is held constant between the knots.

**Figure 2:**
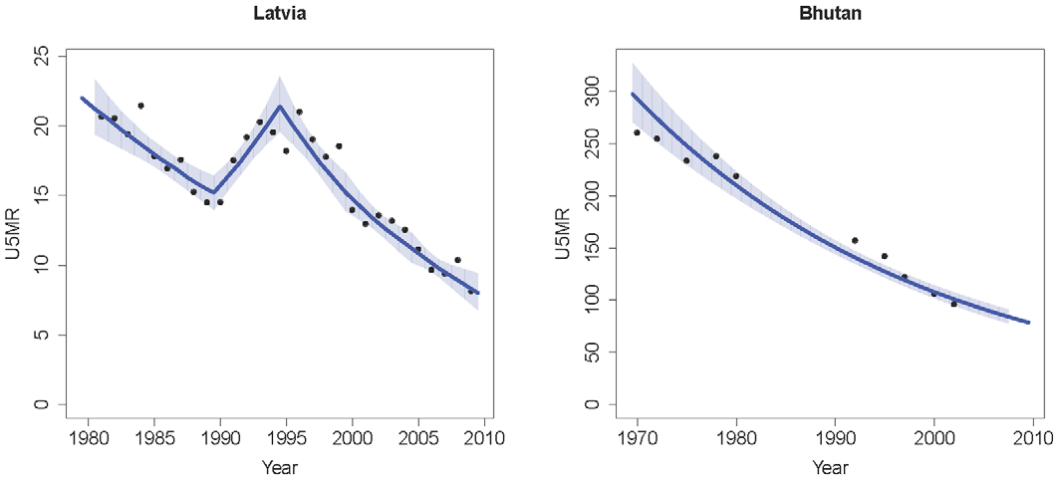
Spline estimates for Latvia and Bhutan. Spline estimates of U5MR in blue, observations in black, 95% confidence intervals are represented by the blue area.

The drawback of the spline approach is that it leads to inaccuracies in the estimated rate of decline over time by forcing it to be constant between the knots, especially when there is a large difference between the rate of decline from one period to the next, or when the rate of decline is kept constant during a long observation period. This is illustrated in Figure 2 for the Latvia. Under-five mortality might have increased in early 1990s, but there is no evidence in the data for the “elbows” in the U5MR estimates in 1989 and 1994: the abrupt changes in the rate of decline are caused by the placements of the knots. If the knots would have been placed a few years earlier or later, a different trend in U5MR would have been estimated.

The spline estimates for Bhutan are illustrated in the second plot of Figure 2. Because of the limited number of observations, the rate of decline is kept constant throughout the observation period in Bhutan. As a result of this approach, the U5MR estimates do not follow the trend in the data in the early 1990s very well. Moreover, the estimation approach gives relatively narrow confidence bounds for U5MR during the 1980s, even though data during that period are lacking.

To overcome the drawbacks of using a piece-wise constant model for the rate of decline in the spline estimation approach, we developed an alternative method for estimating under-5 mortality for all countries, which allows the rate of decline to change continuously over time. A Bayesian hierarchical model is used to estimate the model parameters for each country, such that trends within countries are estimated based on the country's own history as well as observed trends in all other countries.

## Methods

### Time series model

**Figure 3:**
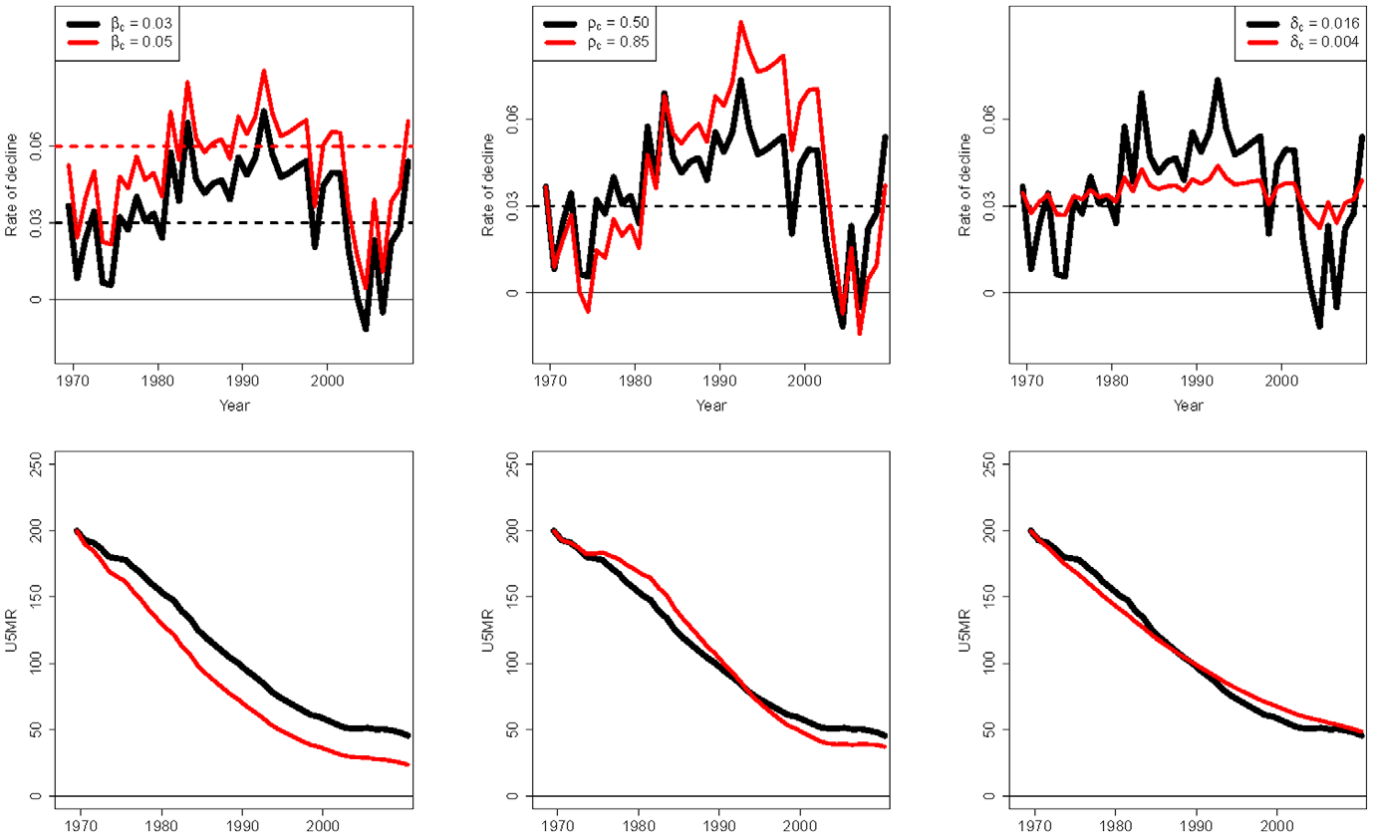
Model illustration. Simulations of the annual rate of decline and the corresponding trajectories of U5MR for different values of average rate of decline 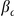, autoregressive parameter 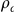 and variance parameter 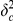. The start level of U5MR is set at 200 deaths per 1,000 live births in 1970. In all plots the black curve represents one simulated trajectory for 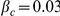, 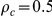 and 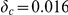. The red curve illustrates the effect of changes in one of the parameters. Column 1: A larger average decline 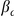 gives a faster decline in U5MR. Column 2: For outcomes of 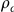 that are closer to one, the rates of decline are more correlated over time and the trajectory of 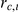 can “stay away from” the average decline during a longer period. Column 3: Decreasing variance parameter 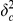 gives a smoother trend in the rate of decline and U5MR.

The rate of decline in U5MR is modeled with a time series model, such that changes are not limited to occur at predefined time points. For country 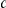, the rate of decline from year 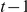 to year 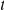 is given by 
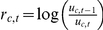
, where 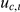 is the U5MR in year 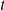. We assume that the rate of decline fluctuates around a country-specific average annual change using an autoregressive time series model of order one (an AR(1) model). For each country, the parameters of the time series model are (i) average rate of decline 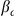, (ii) parameter 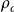 that models the autocorrelation in the rate of decline, and (iii) a variance parameter 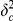 which determines the smoothness of its trajectory. The AR(1) model is given by:
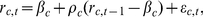
(1)

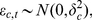
(2)where 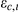 are the distortion terms that model the random fluctuations in 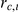 during observation year 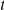. The model behavior is illustrated in Figure 3. In the top row the black curve represents one simulated trajectory of the annual rate of decline for 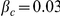, 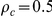 and 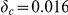, the bottom row illustrates the corresponding trajectory of U5MR based on a start level of 200 deaths per 1,000 births in 1970. The parameters are changed individually in each plot to illustrate their influence on the rate of decline and corresponding U5MR trajectory (the standardized distortion terms 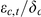 in the time series model are held constant, and values for 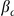, 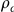 and 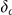 are chosen for illustrative purpose, within the range of the country-specific estimates). The influence of the average decline 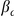 is shown in the first column: a larger rate of decline gives a faster decline in U5MR. For outcomes of 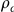 that are closer to one, the rates of decline are more correlated over time and the trajectory of 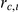 can “stay away from” the average decline during a longer period. This effect is illustrated in the second column of Figure 3 where 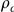 is increased from 0.5 to 0.85. The resulting rate of decline in red is larger than 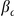 from 1981 until 2003, leading to a faster decline of U5MR during that period. Finally, decreasing variance parameter 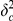 gives a smoother trend in the rate of decline and U5MR, as illustrated in the third column. The simulations illustrate the interpretation of the parameters and the flexibility of the model to represent various trends in the rate of decline and U5MR.

### Data model

The sampling model for the data is taken from the IGME spline estimation approach such that the U5MR estimates from the two modeling approaches are directly comparable. The sampling model is given by:
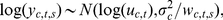
(3)where 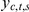 is the observed U5MR for country 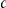, year 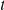, observation 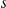. Its variance on the log-scale is proportional to the inverse of its weight 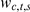, that quantifies its relative accuracy (as discussed in the Introduction). The country-specific error variance is denoted by 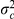. Observation years are rounded and observations with zero weight are excluded.

### Bayesian hierarchical model

Estimating the country-specific parameters 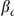 and 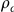 presents a challenge for some countries because of a limited number of observations and/or “noisy data” (large sampling and non-sampling errors). We use a Bayesian hierarchical model [5], [6] to estimate these parameters in each country, such that the estimates for 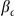 and 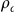 are based on the observed trends in the country of interest, as well as observed trends in other countries. For example, based on the small number of observations in Bhutan, it is not clear how much the rate of decline could have fluctuated around the observed average rate of decline during the late 1980s; there is limited to no information about autocorrelation parameter 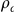 in the time series model for Bhutan. In the hierarchical modeling approach, information about the extent of the fluctuations, and thus parameter 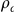, is obtained from other countries with more observations, where trends have been observed in more detail, such as Pakistan or Latvia.

In the Bayesian hierarchical model for average rate of decline 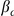 we assume that for all countries 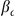 is drawn from a probability distribution that represents the range of outcomes of the average annual growth across all countries. For 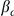 in a specific country, its probability distribution based on the world-level experience is then updated using Bayes' theorem with the observed trend in the country, which results in the posterior distribution for 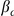. The resulting estimates (draws from the posterior distribution) can be viewed as weighted averages of a “world average rate of decline” and information from the country data: the fewer the number of observations in the country of interest, the more its estimates of 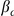 are shrunk towards the estimates in other countries. The hierarchical distribution for 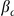 is given by:
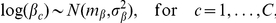
where 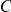 is the total number of countries (here 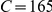), and hierarchical (world-level) mean and variance denoted by 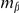 and 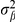. The log-transform is applied to restrict the average rate of decline to be positive. The hierarchical mean and variance are added as parameters to the model, and estimated based on observed rates of declines in all countries.

The same approach is used for the autoregressive parameter 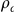. Its hierarchical distribution is given by:
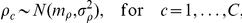
with 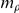 the hierarchical mean and 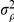 the variance of the 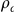
's.

Variance parameter 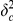 determines the smoothness of the trajectories: a larger variance allows trajectories from the time series model to follow trends in the data more closely. Because error variance is estimated within each country separately, an upper bound on 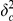 is needed to avoid highly fluctuating U5MR trajectories that follow the data closely in countries where large measurement errors are present. Smoothness of trajectories is guaranteed by restricting the expected absolute outcome of the annual distortion term to be smaller than the average rate of decline 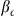, which corresponds to 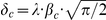, with 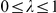.

### Estimation

The under-5 mortality rate was estimated with the Bayesian hierarchical time series model for 165 countries within their observation periods, and projected for an additional five years after the most recent observation year in each country. All model parameters were estimated in a Bayesian framework. Diffuse prior distributions were assigned to the additional model parameters. The complete model and assigned prior distributions are given in Supporting Information S1. A Markov Chain Monte Carlo (MCMC) algorithm was used to sample from the posterior distribution of the parameters using Winbugs software [7], [8]. Results were obtained from eight chains; the total number of iterations in each chain was 600,000, keeping every 500th iteration, and discarding the first 100,000 iterations as burn-in. Convergence of all model parameters and log(U5MR) was assessed visually using trace plots and with the run length diagnostic of Raftery and Lewis [9], [10]. This resulted in a posterior sample of U5MR trajectories for each country. The “best” estimates during the observation period were given by the median outcome, and 95% credible intervals by the 2.5% and 97.5% percentiles of the set of outcomes in each year. Similarly for the projection period, the “best” projection was given by the median outcome, and the 2.5% and 97.5% percentiles were used to construct 95% projection intervals.

### Model validation

Cross-validation was used to validate model performance, in which some observations are excluded while the method is applied to the remaining observations (the training set). Calibration was assessed based on the predictive distributions for the excluded observations by checking that the prediction intervals contain the left-out observations the right proportion of the time. The proportion of excluded observations that fall outside their 80% and 95% prediction intervals was used as a measure of calibration. These proportions are expected be around 20% and 5%, respectively. Higher proportions suggest that the credible and projection intervals for U5MR are too narrow, while smaller proportions suggest that the credible and projection intervals for U5MR are too wide.

When leaving out observations, the structure of the data should be taken into account, e.g. by leaving out all observations from the same survey, such that the observations in the test set are independent of the observations in the training set. Unfortunately the data on *IGME info* are not yet in a format which allows for this exercise to be carried out in an automated fashion. Instead, we carried out two validation exercises. In the first exercise, 20% of the observations within each country are left out at random, such that the training set consists of 80% of all observations. In the second validation exercise, all observations in the last five years of the observation period within each country were left out. That is, if the last observation year is 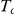 in country 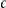, all observations in years 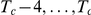 were excluded from the training set for that country. The motivation for this second exercise is to check the predictive performance of the model: for many countries the last observation year is well before 2010, thus the “2010 estimates” are in fact projections.

To assess how confident one can be about the current estimates and projections, we also compared the current estimates and projections of U5MR based on the full data set to the credible and projection intervals for U5MR based on the training set. The goal is to check that additional data has not changed the U5MR estimates and projections significantly: as more data become available, we expect the current (updated) estimates to lie well within the previously constructed credible and projection intervals. The smaller the proportion of estimates and projections that fall outside their respective credible and projection intervals, the better (contrary to the previously described measures of calibration).

## Results

### U5MR estimates

**Figure 4:**
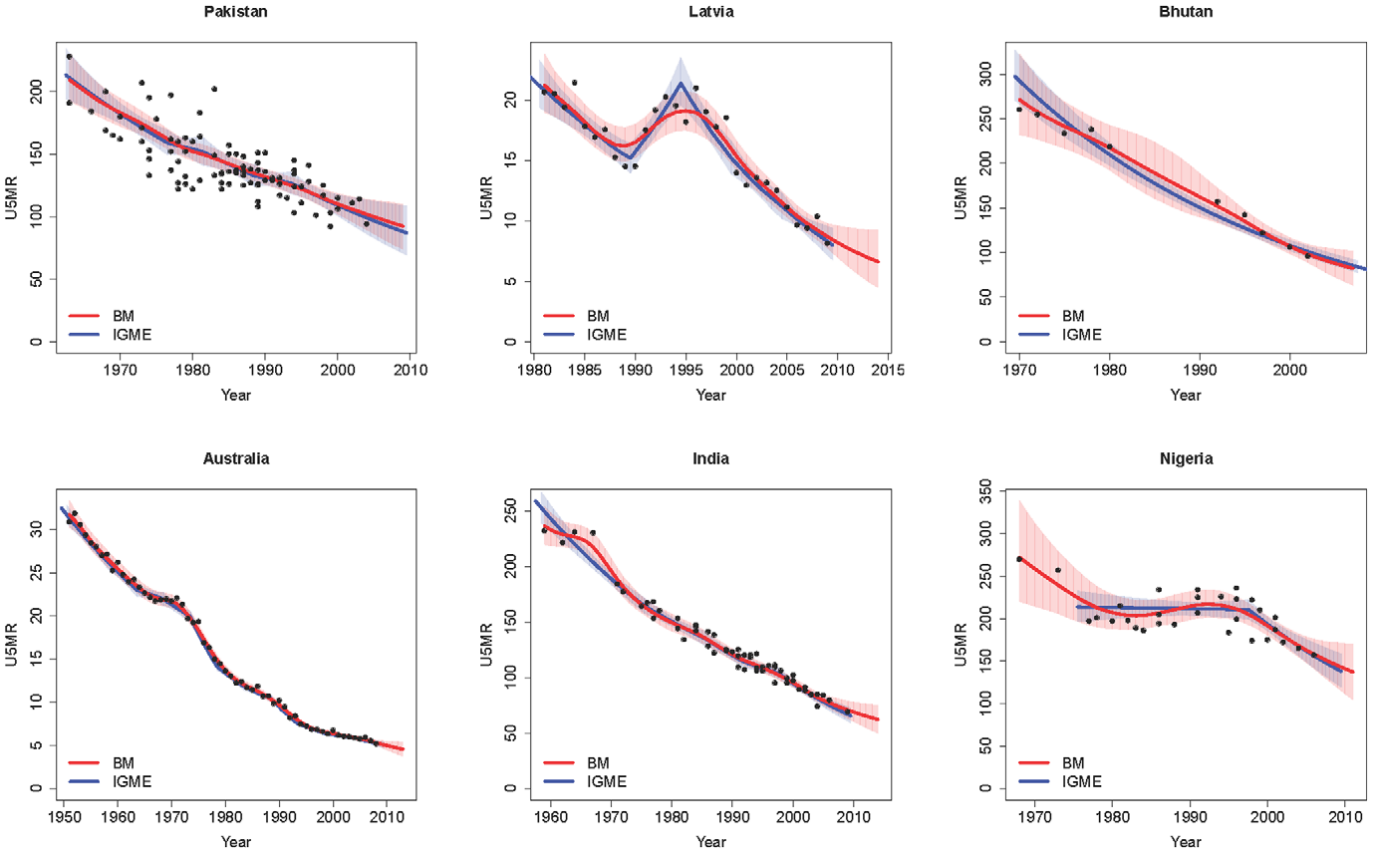
Estimates of U5MR for Pakistan, Latvia, Bhutan, Australia, India and Nigeria. The median U5MR estimates from the Bayesian model (BM) are shown in red, and the IGME estimates are shown in blue. The 95% credible/confidence and projection intervals are represented by the red area for the Bayesian model, and the blue area for the IGME estimates. Observations are represented by the black dots.

The under-5 mortality rate was estimated with the Bayesian hierarchical time series model (BM) for 165 countries within their observation periods, and projected for an additional five years after the most recent observation year in each country. For many countries, the differences between the spline estimates and the median estimates of the BM are small. This is illustrated in Figure 4 for Pakistan. The median estimates from the BM are given by the red line, and the red shaded area represents the 95% credible and projection intervals. The IGME estimates are shown in blue, with the 95% confidence and projection intervals represented by the blue shaded area. For Pakistan, the intervals overlap and point estimates are similar.

As discussed in the introduction, the limited number of knots in the spline approach introduces abrupt changes in the rate of decline in U5MR in Latvia. The BM estimates differ from the IGME estimates, as shown in Figure 4: U5MR changes more smoothly in the BM estimates. For Bhutan, a country with a small number of observations, the spline regression approach gives U5MR estimates that do not follow the trend in the data in the early 1990s very well, and lead to relatively narrow confidence bounds for U5MR during the 1980s, even though data during that period are lacking. The BM estimates are closer to the trend in the data in the 1990s than the spline results; the estimated standard deviation 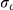 from Eq.3 for Bhutan is 2.5 for the BM estimates, compared to 5.6 for the spline estimates (the estimates for 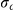 were calculated using the sample of the differences between the observations and the estimates on the log-scale, accounting for the weights). The BM credible bounds for Bhutan differ from the spline confidence bounds and show greater uncertainty about the U5MR in the 1980s than estimated with the spline approach.

The results for Australia, India and Nigeria are shown in Figure 4 on the second row. The results with the BM are very similar to the results from the spline approach for Australia, an example of a country with a high quality vital registration system; the BM estimates follow the observations closely and there is little uncertainty about the level of U5MR. Based on the spline estimates, India and Nigeria together account for nearly a third of all under-five deaths worldwide in 2009 [11]. The BM estimates are similar to the spline estimates for these countries but exhibit greater uncertainty in 2009.

The results for all countries are given in Supporting Information S1. For 73% of all countries, including all countries in Figure 4, the estimated country-specific error standard deviation 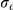 is smaller based on the BM estimates than for the spline estimates, which means that the BM estimates are closer to the data than the spline estimates (taking into account the data weights) for the majority of all countries.

### Rate of decline

**Figure 5:**
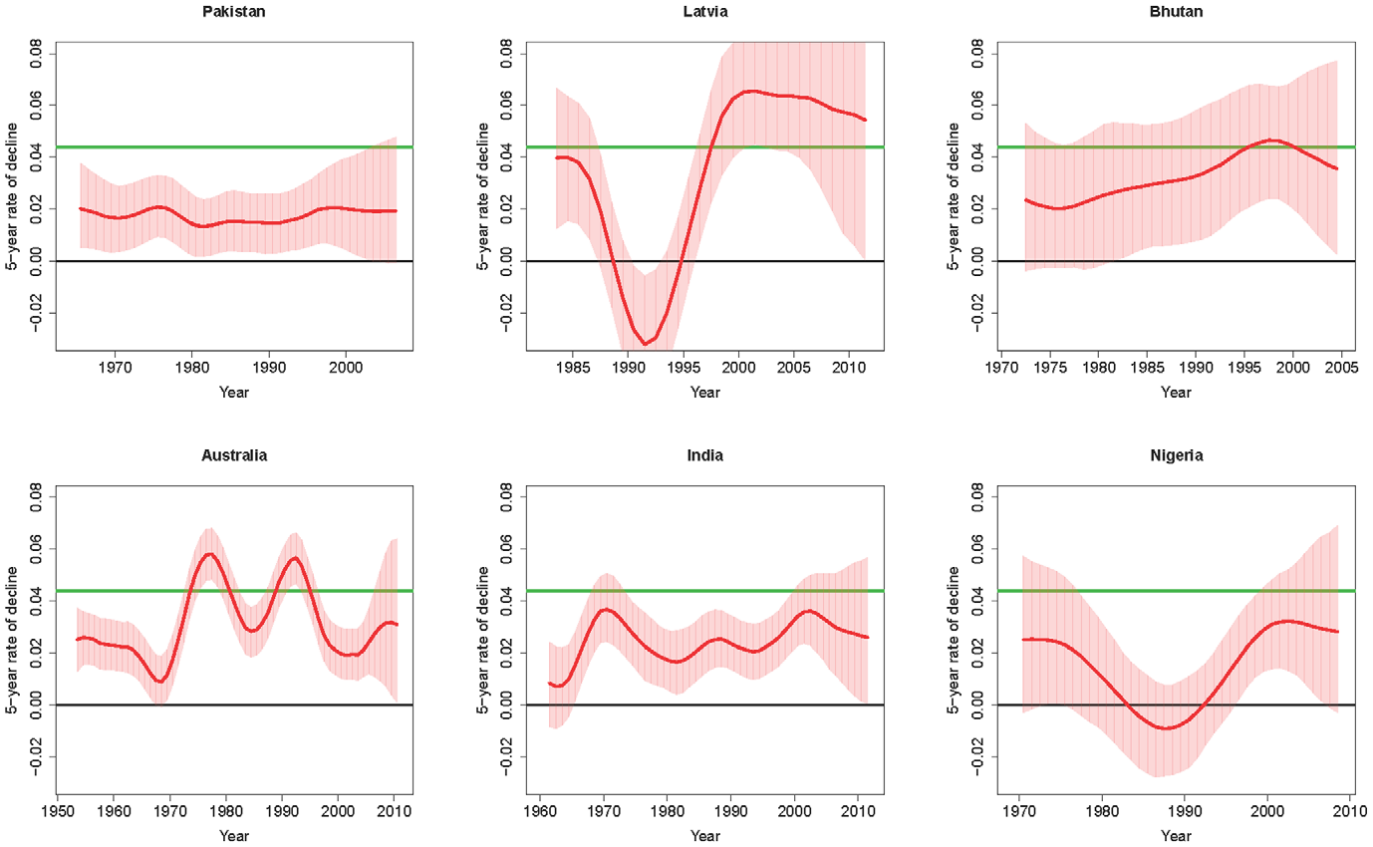
Estimates of the annual rate of decline for Pakistan, Latvia, Bhutan, Australia, India and Nigeria. The average rate of decline over a 5-year period is plotted against the midpoint of the 5-year period under consideration. The median estimates are shown in red, and the 95% credible/confidence and projection intervals are represented by the red area. The green line is drawn at a rate of decline of 4.4%, which corresponds to Millennium Development Goal 4.

Millennium Development Goal 4 calls for a reduction in the under-five mortality rate by two-thirds between 1990 and 2015, which corresponds to an annual rate of decline of 4.4%. This target has increased the attention for monitoring the rate of decline in U5MR to assess whether countries are reducing U5MR at the 4.4% rate a year, and whether the rate of decline has increased recently in countries where progress has been slow. The average annual rate of decline, measured over 5-year periods, is plotted for Pakistan, Latvia, Bhutan, Australia, India and Nigeria in Figure 5. The green line is drawn at the target rate of 4.4%. The median estimates for the rate of decline have been above 4.4% for a short period in Bhutan, and for low-mortality countries Australia and Latvia; on average, the rate of decline in the countries with higher mortality rates tends to be much lower.

**Figure 6:**
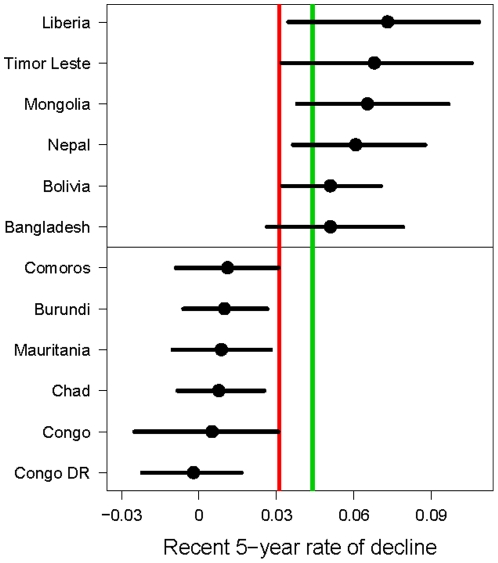
Estimates of the recent 5-year rate of decline for the six high mortality countries with the highest/lowest outcomes. Median estimates (dots) and 95% credible intervals (horizontal line) for the rate of decline in the last five observation years in the six countries with the highest, and the six countries with the lowest median estimate. The red vertical line is the median of all country estimates, which is at 3.5%. The green line is drawn at a rate of decline of 4.4%, which corresponds to Millennium Development Goal 4.

The observed rate of decline in the last five observation years is given in Figure 6 for selected high mortality countries, where high mortality countries are defined as those countries with at least 40 deaths per 1,000 births (as in [1], 55 countries in total). The selected high mortality countries are the six countries with the lowest, and the six countries with the highest estimates of the 5-year rate of decline among all high mortality countries. The highest rates were estimated in Pakistan, Bolivia, Nepal, Mongolia, Timor-Leste and Liberia, while Comoros, Burundi, Mauritania, Chad, Latvia and Congo DR had the lowest rates of decline.

**Figure 7:**
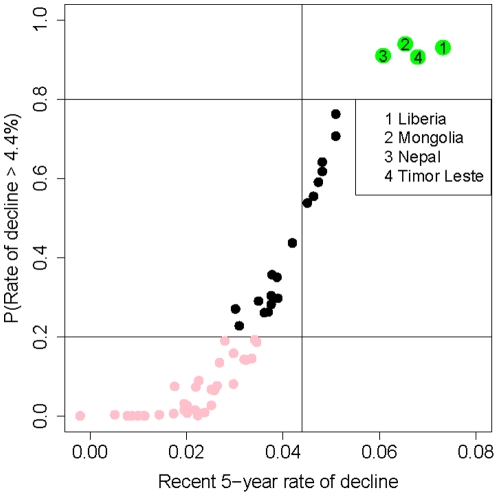
Posterior probability that the recent 5-year rate of decline was larger than 4.4% for all high mortality countries. Probability that the recent 5-year rate of decline in the observation period within each country is more than 4.4%, plotted against the median estimate of its recent rate of decline. The four selected countries in green have a posterior probability of more than 0.8 that the difference was positive. The countries that are plotted in pink have a posterior probability of less than 0.2 that the rate of decline was more than 4.4%.

Bayesian inference allows for calculating the posterior probability (or the posterior odds) that the recent 5-year rate of decline was above the 4.4% goal, which gives more insights into a country's recent progress towards reducing under-five mortality at the target rate. Figure 7 shows the probability that the recent rate of decline was higher than 4.4%, plotted against the median estimate of the rate of decline, for all high mortality countries. The most recent rate of decline was higher than 4.4% with probability 0.8 or higher for only four countries: Liberia, Mongolia, Nepal and Timor-Leste (plotted in green). For almost half of the countries (46%), the posterior probability that the recent rate of decline was less than 4.4% is at least 0.8. This corresponds to posterior odds of at least four to one that the recent rate of decline was less than 4.4% for those countries.

**Figure 8:**
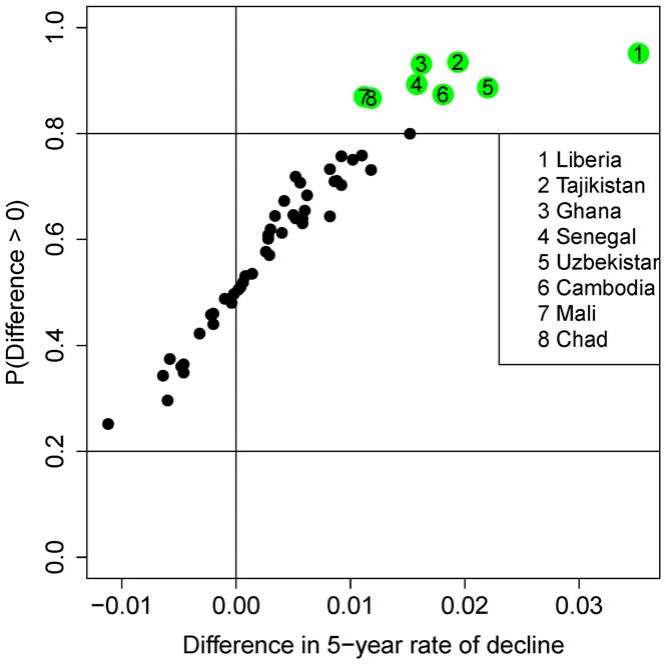
Posterior probability of an increase in the 5-year rate of decline for all high mortality countries. Probability of a positive difference between the most recent 5-year increase and the preceding 5-year increase in the last ten years of the observation period within each country, plotted against the median estimate of that difference. The eight selected countries have a posterior probability of more than 0.8 that the difference was positive.

The difference between the most recent 5-year rate of decline and the preceding 5-year period can be used to assess evidence of acceleration or deceleration in under-five mortality reduction. The difference between the most recent 5-year rate of decline and its preceding 5-year period are shown in Figure 8 for all high mortality countries. The probability of a positive difference, of an increase in the rate of decline, is shown on the vertical axis. For eight countries the posterior odds of a positive increase are at least four to one. These countries are Liberia, Ghana, Tajikistan, Senegal, Uzbekistan, Cambodia, Mali and Chad. There are no countries in which the odds of a negative change in the rate of decline are more than four to one.

### Other model parameters

Histograms of the posterior samples of the non-country specific parameters 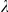, 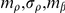 and 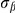 are given in Supporting Information S1. The autocorrelation parameter of the autoregressive process (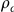) does not vary much between countries: all 95% credible intervals for 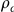 are roughly within 0.5 and 0.9. With 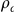 smaller than one for all countries, the rate of decline converges towards to average rate of decline 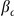 in long term projections, as shown for Pakistan in Figure 5.

**Figure 9:**
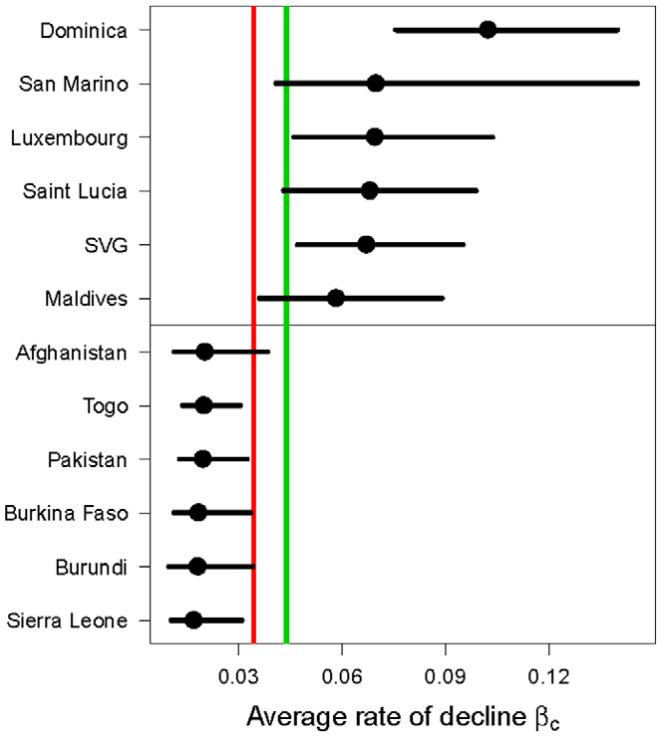
Estimates of the average rate of decline for the six countries with the highest/lowest estimates. Median estimates (dots) and 95% credible intervals (horizontal line) for average rate of decline 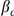 in the six countries with the highest, and the six countries with the lowest median estimate. The red vertical line is the median of all country estimates, which is at 3.4%. The green line is drawn at a rate of decline of 4.4%, which corresponds to Millennium Development Goal 4. (*SVG = St Vincent and the Grenadines).

Figure 9 shows the median estimates and 95% credible intervals for the average rate of decline 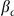 in the six countries with the highest, and the six countries with the lowest median estimates. The red vertical line is the median of all country estimates, which is at 3.4%. The average rates of decline were lowest in Sierra Leone, Burundi, Burkina Faso, Pakistan, Togo and Afghanistan.

### Model validation

To assess model performance we carried out the cross-validation exercises as discussed in the previous section. Countries with less than four observations in the training set were excluded (Korea DPR was excluded in the first exercise in which 20% of the observations were left out at random, and Grenada, Korea DPR, Liechtenstein, Montserrat and Somalia were excluded in the second exercise, where observations during the last five years of the observation period were left out). Detailed results are given in Supporting Information S1.

When leaving out 20% of the observations at random, the results based on the predictive distributions for the left-out observations show that slightly fewer observations fall outside their respective 80% and 95% prediction intervals than expected (13% instead of 20% for the 80% interval, and 4% instead of 5% for the 95% interval), which suggests that the credible bounds for U5MR are somewhat conservative (too wide).

As more data become available, we expect the current (updated) estimates to lie well within the previously constructed credible bounds. This holds true when leaving out 20% of the observations: only 3% of the current estimates fall outside the 80% credible bounds that were constructed based on the training set for the last observation year. Similarly, only 3% of the current projections fall outside the 80% projection intervals that were constructed based on the training set.

When leaving out the observations in the last five observation years within each country, the results for all countries combined suggest that the model is reasonably well calibrated (19% of the left-out observations fall outside their 80% prediction intervals, and 6% of the observations fall outside their 95% prediction intervals). The calibration of the credible interval for the last observation year within the second training set is also satisfactory: few updated estimates fall outside the previously constructed 80% credible intervals for that year. We expect that at most around 20% of the current estimates are outside the previously constructed projection intervals when projecting ahead for five years beyond the last observation year, which holds true for all countries combined: 18% of the estimates fall outside the previously constructed 80% projection intervals.

Broken down by U5MR level, countries with current U5MR between 40 and 100 deaths per 1,000 live births have more observations falling below their respective 80% prediction intervals than expected (19% instead of 10%), which suggests the rate of decline was under-predicted for some countries in this group. This is confirmed when comparing the current estimates for the last observation year to the projection intervals constructed when excluding the last 5 years of data: the current estimates are below the 80% projection intervals for seven out of thirty countries with current U5MR between 40 and 100 deaths per 1,000 live births. This percentage is higher than expected and suggests that the Bayesian hierarchical time series model under-predicted the rate of decline in these seven countries, which are Nepal, Tajikistan, Mongolia, Bangladesh, Bolivia, Ghana and Azerbaijan. The list of countries is not very surprising: the first six countries were highlighted earlier because of either a high most recent 5-year rate of decline or evidence of an increase in the 5-year rate of decline. For all countries, the current estimates are within the previously constructed 95% projection intervals.

## Discussion

We proposed a Bayesian hierarchical time series model for estimating U5MR and constructing short-term projections, as an alternative to the spline regression modeling approach. An autoregressive model for the annual rate of decline in U5MR captures changes more smoothly than a piece-wise linear regression model. The hierarchical approach for estimating the model parameters has the advantage of sharing information about parameter estimates between countries, without restricting these parameters to be equal.

Cross-validation exercises suggest that the proposed model provides U5MR credible bounds which are reasonably well calibrated during the observation period. The out-of-sample performance of the model for all countries combined was satisfactory as well, although the rate of decline was slightly underestimated for countries with a recent acceleration in the rate of decline. This is to be expected: projections quantify “our best guesses” and uncertainty therein based on observed trends in the past. If future trends are significantly different from the past, e.g. through interventions which lead to acceleration in the rate of decline that have not been observed in the past, projection intervals are no longer calibrated. This highlights the need to distinguish between U5MR estimates and projections when analyzing progress towards reducing U5MR: estimates represent observed trends, while projections represent the expected future trends based on the past and model assumptions.

The sampling model for the data in the Bayesian model was taken from the IGME approach such that the U5MR estimates from the two modeling approaches are directly comparable. The observations are assumed unbiased on the log-scale, with variance inversely proportional to an observation-specific weight which has been fixed a priori. Biased observations, such as observations from incomplete vital registration systems, are excluded or down-weighted in the current approach. If biased observations are informative of the trend in U5MR, inclusion of the observation and estimating their biases could improve the estimation of U5MR. The sampling model can also potentially be improved upon by including error variance parameters for different data types which are estimated from the data, and by using a hierarchical model to exchange information between countries about measurement errors. Sharing information about measurement errors between countries would give more informed estimates of error variance in countries with fewer observations, and it would remove the need for the restriction on the variance of the distortion terms in the current proposed model.

For countries with high HIV/AIDS prevalence and/or extreme events, IGME uses a loess smoother to model U5MR [2], [11]–[13]. The main difficulties in the loess smoother approach are the choice of the smoothing parameter and the construction of confidence intervals. In IGME's 2010 revision of the U5MR estimates, the smoothing parameter was determined based on the number of independent data series within a country [2]. Confidence bounds were not included for the set of countries in which the loess smoother was used. The Bayesian modeling approach could also be used for these countries, although adjusted modeling procedures might be needed for the periods with extreme events or to model reversal in trends in high HIV prevalence countries.

Gaussian process regression (GPR) has been proposed as an alternative modeling approach for estimating U5MR for all countries [14]. In this modeling approach, trajectories of U5MR on the log(10)-scale are draws from a multivariate normal distribution. The prior mean is given by a loess smoother fit to the data. The U5MR estimates from our proposed model cannot be compared directly to the GPR's estimates because the data set that was used in [14] differs from the data set from *IGME info*. In-sample and out-of-sample validation checks were used to assess model performance of the GPR model, but the reported measures of calibration only assessed how close the U5MR estimates based on the training set were to the left-out data from the test set. The widths of the reported uncertainty bounds from the GPR approach have not been validated. We found that uncertainty in U5MR estimates and its rate of decline is non-negligible for many countries and needs to be taken into account when analyzing progress towards MDG4. For this reason, validation exercises of the uncertainty bounds of any U5MR modeling approach should be included in model validation exercises, such that results can be compared across models.

## Supporting Information

Supporting Information S1 **Estimating the Under-Five Mortality Rate Using a Bayesian Hierarchical Time Series Model.**
(PDF)
