# Supplementary material for: Estimating the Under-Five Mortality Rate Using a Bayesian Hierarchical Time Series Model
Source: PLoS One. 2011 Sep 28;6(9):e23954. doi: 10.1371/journal.pone.0023954 (PMC3182159; doi:10.1371/journal.pone.0023954)
Supplement: Supporting Information S1 — Estimating the Under-Five Mortality Rate Using a Bayesian Hierarchical Time Series Model. (PDF) [file pone.0023954.s001.pdf]

Supporting Information S1 for:

Estimating the Under-Five Mortality Rate Using a Bayesian Hierarchical Time Series Model

Leontine Alkema*, Wei Ling Ann

Department of Statistics and Applied Probability, National University of Singapore, Singapore.

*E-mail: alkema@nus.edu.sg

Bayesian hierarchical time series model

The Bayesian hierarchical time series model is given by (with S_c the first observation year for country c):

$$
\begin{aligned}
r_{c,t} &= \log\left(\frac{u_{c,t-1}}{u_{c,t}}\right), \text{ for } t \geq S_c, \\
r_{c,t} &= \beta_c + \rho_c(r_{c,t-1} - \beta_c) + \varepsilon_{c,t}, \text{ for } t \geq S_c, \\
\varepsilon_{c,t} &\sim N(0, \delta_c^2), \\
\log(u_{c,S_c-1}) &\sim N(\log(y_{c,S_c,1}), 1), \\
r_{c,S_c-1} &\sim N\left(\beta_c, \frac{\delta_c^2}{1-\rho_c^2}\right), \\
\log(\beta_c) &\sim N(m_\beta, \sigma_\beta^2), \\
m_\beta &\sim N(0, 10^2), \\
1/\sigma_\beta^2 &\sim \text{Gamma}(0.01, 0.001), \\
\rho_c &\sim N(m_\rho, \sigma_\rho^2), \\
m_\rho &\sim N(0, 10^2), \\
1/\sigma_\rho^2 &\sim \text{Gamma}(0.01, 0.01), \\
\delta_c &= \lambda \cdot \beta_c \cdot \sqrt{\pi/2}, \\
\lambda &\sim U(0, 1), \\
\log(y_{c,t,s}) &\sim N(\log(u_{c,t}), \sigma_c^2/w_{c,t,s}), \\
1/\sigma_c^2 &\sim \text{Gamma}(0.01, 0.01).
\end{aligned}
$$

Histograms of the posterior samples of the non-country specific parameters λ, $m_\rho, \sigma_\rho, m_\beta$ and σ_β are given in Figure 1. The estimates for all countries are given in Figure 2.

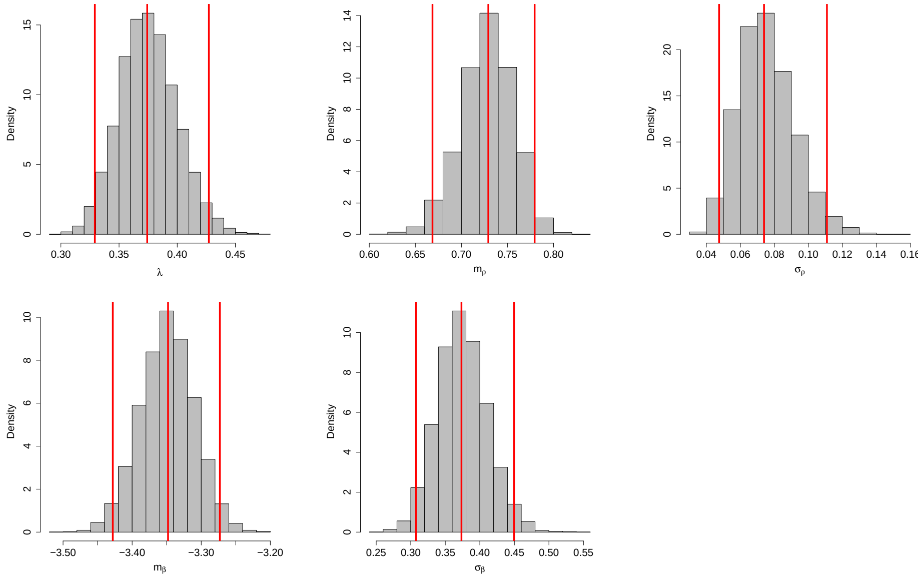

Figure 1. Histograms of the posterior samples of λ, $m_\rho, \sigma_\rho, m_\beta$ and σ_β. The 95% credible intervals and median estimates are represented by the red lines.

Results of the validation exercises

Table 1. Cross-validation results when leaving out 20% of the observations.

Observations	# Obs	Above median	Below 80% PI	Above 80% PI	Below 95% PI	Above 95% PI
All	1310	0.46	0.06	0.07	0.02	0.02
U5MR $<$ 40	931	0.46	0.07	0.08	0.02	0.02
U5MR $\geq$ 40	379	0.45	0.06	0.07	0.02	0.01
40 $\leq$ U5MR $<$100	235	0.44	0.06	0.07	0.02	0.01
U5MR $\geq$ 100	144	0.47	0.04	0.06	0.01	0.01
**Expected proportions**		**0.50**	**0.10**	**0.10**	**0.025**	**0.025**

The proportion of excluded observations that fall above the median U5MR estimate and outside their 80% and 95% prediction intervals (PI), when leaving out 20% of the observations at random. The results are broken down by the median estimate of the U5MR level in the most recent observation year within the country (based on the complete data set).

Table 2. Change in U5MR estimate in last observation year when leaving out 20% of the observations.

Countries	# Countries	Above median	Below 80% CI	Above 80% CI	Below 95% CI	Above 95% CI
All	164	0.48	0.02	0.01	0.01	0.00
U5MR $<$ 40	110	0.42	0.03	0.02	0.02	0.00
U5MR $\geq$ 40	54	0.59	0.02	0.00	0.00	0.00
40 $\leq$ U5MR $<$100	30	0.57	0.03	0.00	0.00	0.00
U5MR $\geq$ 100	24	0.62	0.00	0.00	0.00	0.00
**Expected proportions**		**0.50**	$\leq$ **0.10**	$\leq$ **0.10**	$\leq$ **0.025**	$\leq$ **0.025**

The proportion of countries in which the median U5MR estimate for the last observation year based on the full data set falls above the median estimate and outside the 80% and 95% credible intervals (CI), that were constructed based on a training data set in which 20% of the observations were left out. The results are broken down by the median estimate of the U5MR level in the most recent observation year within the country based on the complete data set. Smaller outcomes suggest better calibration for the expected proportions that are denoted with $\leq$ 0.10 and 0.025.

Table 3. Change in 5-year U5MR projection when leaving out 20% of the observations.

Countries	# Countries	Above median	Below 80% PI	Above 80% PI	Below 95% PI	Above 95% PI
All	164	0.54	0.02	0.01	0.00	0.00
U5MR $<$ 40	110	0.44	0.03	0.01	0.00	0.00
U5MR $\geq$ 40	54	0.74	0.02	0.00	0.00	0.00
40 $\leq$ U5MR $<$100	30	0.73	0.03	0.00	0.00	0.00
U5MR $\geq$ 100	24	0.75	0.00	0.00	0.00	0.00
**Expected proportions**		**0.50**	$\leq$ **0.10**	$\leq$ **0.10**	$\leq$ **0.025**	$\leq$ **0.025**

The proportion of countries in which the 5-year median U5MR projection based on the full data set falls above the median projection and outside the 80% and 95% projection intervals (PI), which were constructed based on a training data set in which 20% of the observations were left out. The results are broken down by the median estimate of the U5MR level in the most recent observation year within the country based on the complete data set. Smaller outcomes suggest better calibration for the expected proportions that are denoted with $\leq$ 0.10 and 0.025.

Table 4. Cross-validation results when leaving out the last five observation years.

Observations	# Obs	Above median	Below 80% PI	Above 80% PI	Below 95% PI	Above 95% PI
All	631	0.48	0.10	0.09	0.03	0.03
U5MR $<$ 40	461	0.51	0.08	0.10	0.03	0.03
U5MR $\geq$ 40	170	0.42	0.14	0.05	0.02	0.02
40 $\leq$ U5MR $<$100	98	0.38	0.19	0.03	0.03	0.01
U5MR $\geq$ 100	72	0.49	0.07	0.08	0.01	0.03
**Expected proportions**		**0.50**	**0.10**	**0.10**	**0.025**	**0.025**

The proportion of excluded observations that fall above the median U5MR estimate and outside their 80% and 95% prediction intervals (PI), when leaving out the most recent five years of the observation period. The results are broken down by the median estimate of the U5MR level in the most recent observation year within the country (based on the complete data set).

Table 5. Change in U5MR estimate in last observation year when leaving out the last five observation years.

Countries	# Countries	Above median	Below 80% CI	Above 80% CI	Below 95% CI	Above 95% CI
All	160	0.45	0.06	0.04	0.00	0.01
U5MR $<$ 40	107	0.50	0.07	0.06	0.00	0.01
U5MR $\geq$ 40	53	0.36	0.04	0.00	0.00	0.00
40 $\leq$ U5MR $<$100	30	0.27	0.03	0.00	0.00	0.00
U5MR $\geq$ 100	23	0.48	0.04	0.00	0.00	0.00
**Expected proportions**		**0.50**	$\leq$ **0.10**	$\leq$ **0.10**	$\leq$ **0.025**	$\leq$ **0.025**

The proportion of countries in which the median U5MR estimate based on the full data set falls above the median estimate and outside the 80% and 95% credible intervals (CI) in the last observation year in the training set, where the credible intervals were constructed based on the training set in which the most recent five years of the observation period were left out. The results are broken down by the median estimate of the U5MR level in the most recent observation year within the country based on the complete data set. Smaller outcomes suggest better calibration for the expected proportions that are denoted with $\leq$ 0.10 and 0.025.

Table 6. Change in 5-year U5MR projection when leaving out the last five observation years.

Countries	# Countries	Above median	Below 80% PI	Above 80% PI	Below 95% PI	Above 95% PI
All	160	0.46	0.11	0.07	0.04	0.01
U5MR $<$ 40	107	0.51	0.09	0.10	0.05	0.02
U5MR $\geq$ 40	53	0.34	0.15	0.02	0.02	0.00
40 $\leq$ U5MR $<$100	30	0.27	0.23	0.00	0.00	0.00
U5MR $\geq$ 100	23	0.43	0.04	0.04	0.04	0.00
**Expected proportions**		**0.50**	$\leq$ **0.10**	$\leq$ **0.10**	$\leq$ **0.025**	$\leq$ **0.025**

The proportion of countries in which the median U5MR estimate for the last observation year based on the full data set falls above the median projection and outside the 80% and 95% projection intervals (PI), that were constructed based on a training data set in which observation in the last five observation years were left out. The results are broken down by the median estimate of the U5MR level in the most recent observation year within the country based on the complete data set. Smaller outcomes suggest better calibration for the expected proportions that are denoted with $\leq$ 0.10 and 0.025.

Figure 2. U5MR estimates for all countries (in alphabetical order). The median estimates from the Bayesian model (BM) are shown in red, and the IGME estimates are shown in blue. The 95% credible/confidence and projection intervals are represented by the red area for the Bayesian model, and the blue area for the IGME estimates. Observations are represented by the black dots.

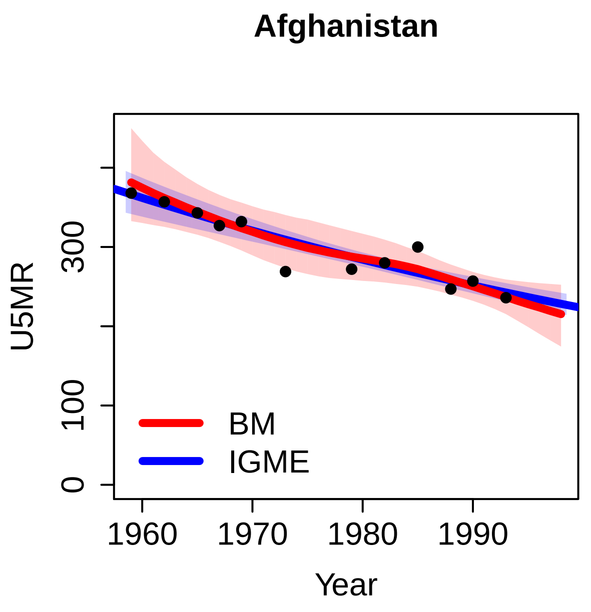
Afghanistan
U5MR
0
100
300
1960
1970
1980
1990
Year
BM
IGME
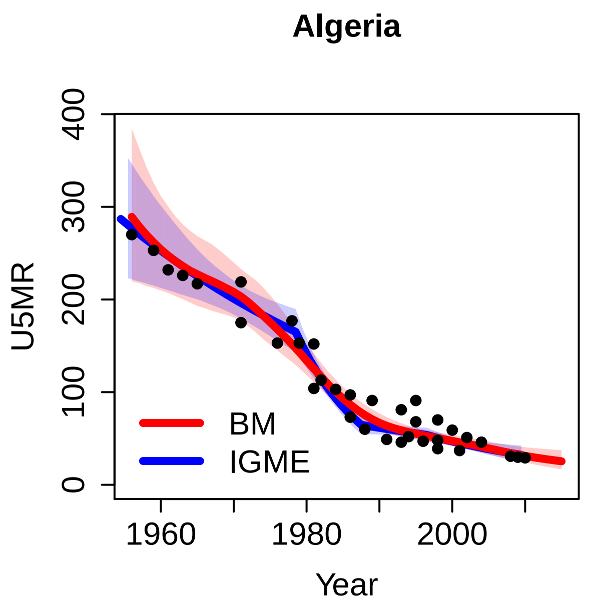
Algeria
U5MR
0
100
200
300
400
1960
1980
2000
Year
BM
IGME
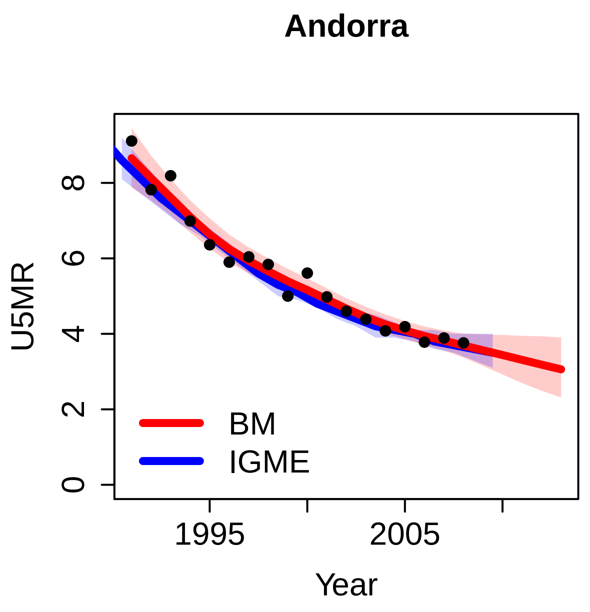
Andorra
U5MR
0
2
4
6
8
1995
2005
Year
BM
IGME
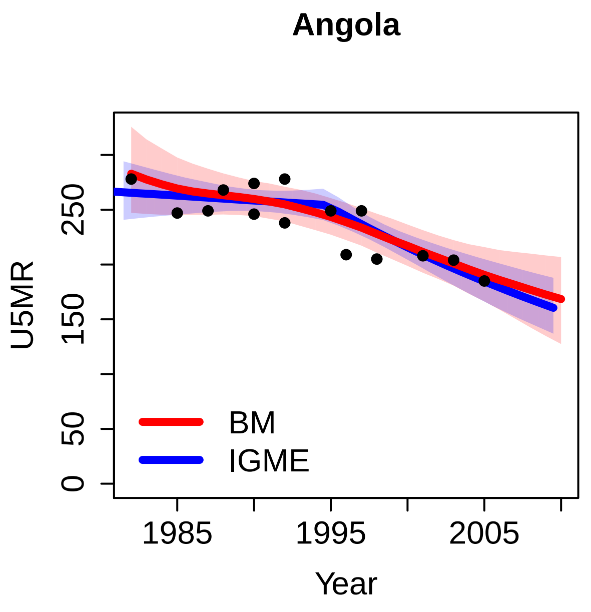
Angola
U5MR
0
50
150
250
1985
1995
2005
Year
BM
IGME
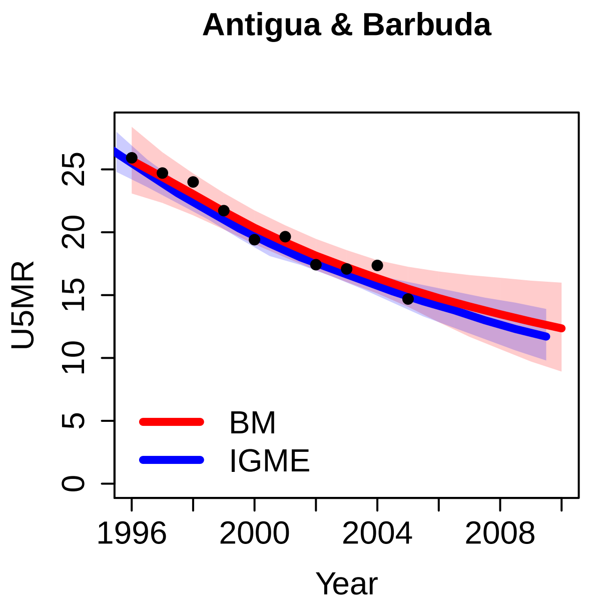
Antigua & Barbuda
U5MR
0
5
10
15
20
25
1996
2000
2004
2008
Year
BM
IGME
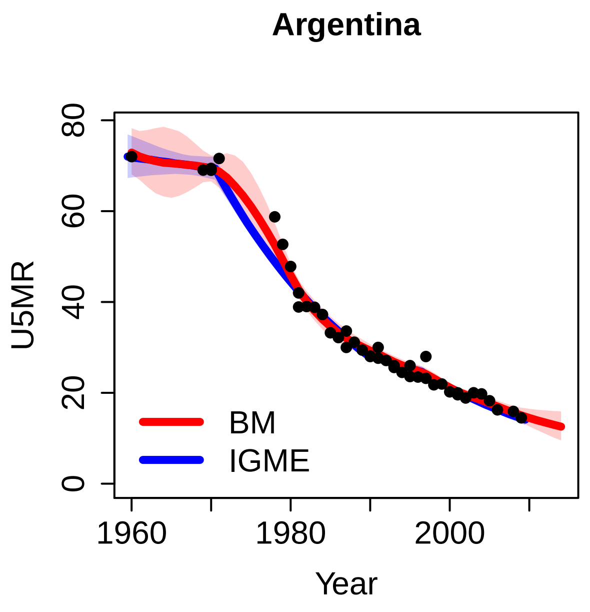
Argentina
U5MR
0
20
40
60
80
1960
1980
2000
Year
BM
IGME
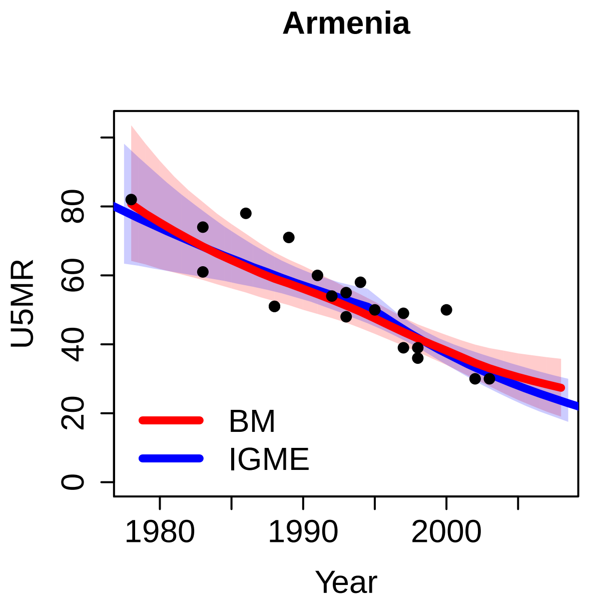
Armenia
U5MR
0
20
40
60
80
1980
1990
2000
Year
BM
IGME
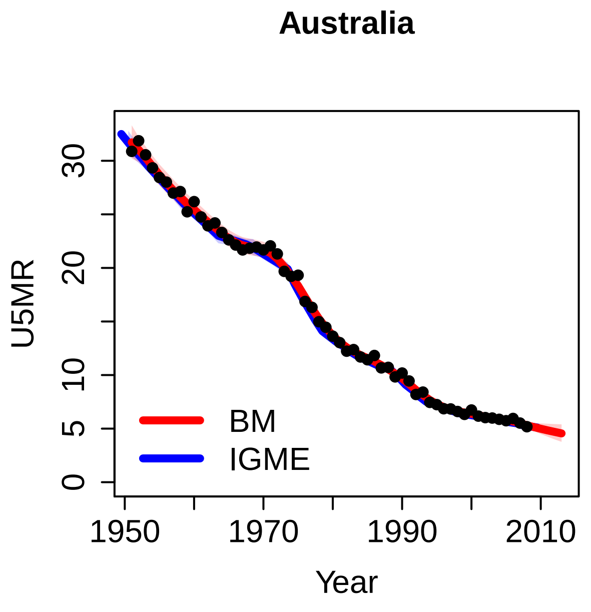
Australia
U5MR
0
5
10
20
30
1950
1970
1990
2010
Year
BM
IGME
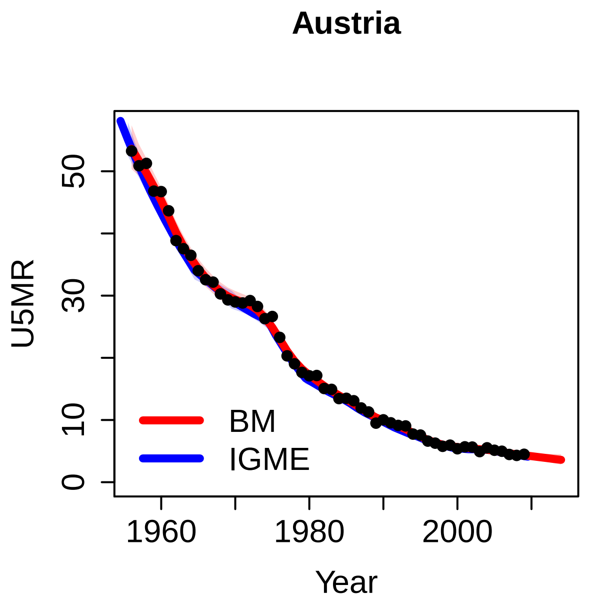
Austria
U5MR
0
10
30
50
1960
1980
2000
Year
BM
IGME
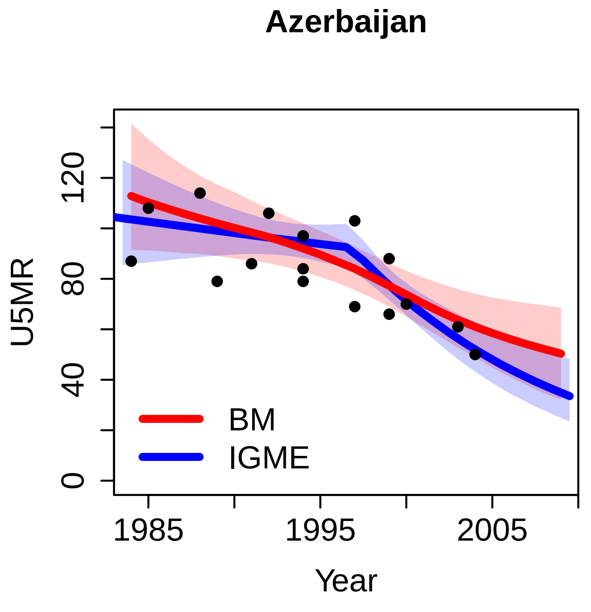
Azerbaijan
U5MR
0
40
80
120
1985
1995
2005
Year
BM
IGME
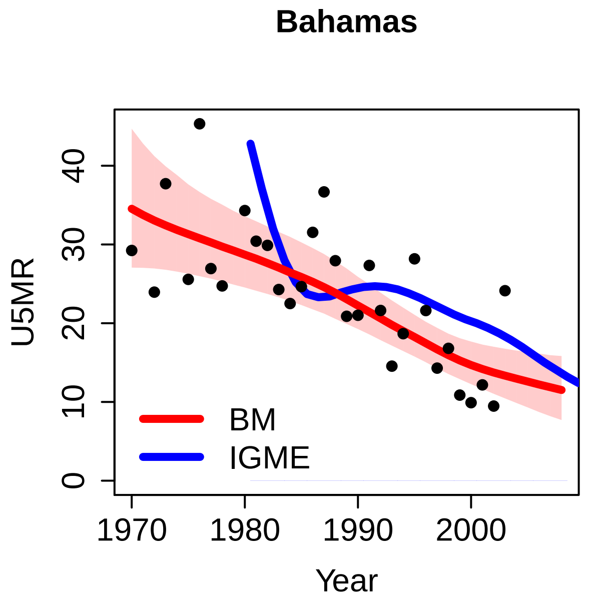
Bahamas
U5MR
0
10
20
30
40
1970
1980
1990
2000
Year
BM
IGME
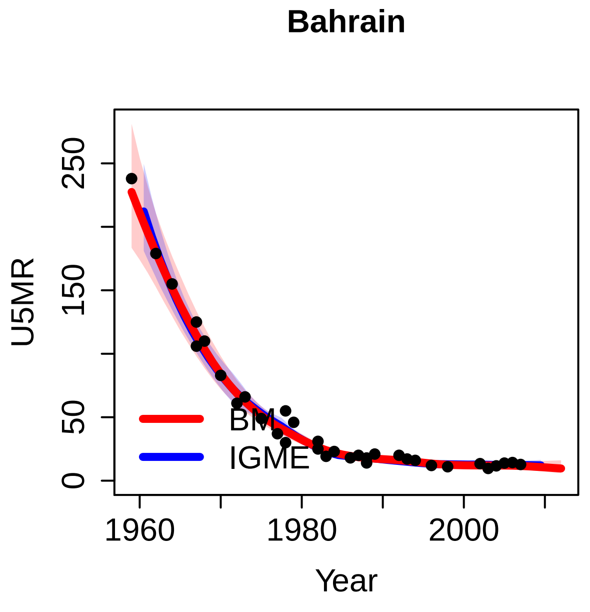
Bahrain
U5MR
0
50
150
250
1960
1980
2000
Year
BM
IGME

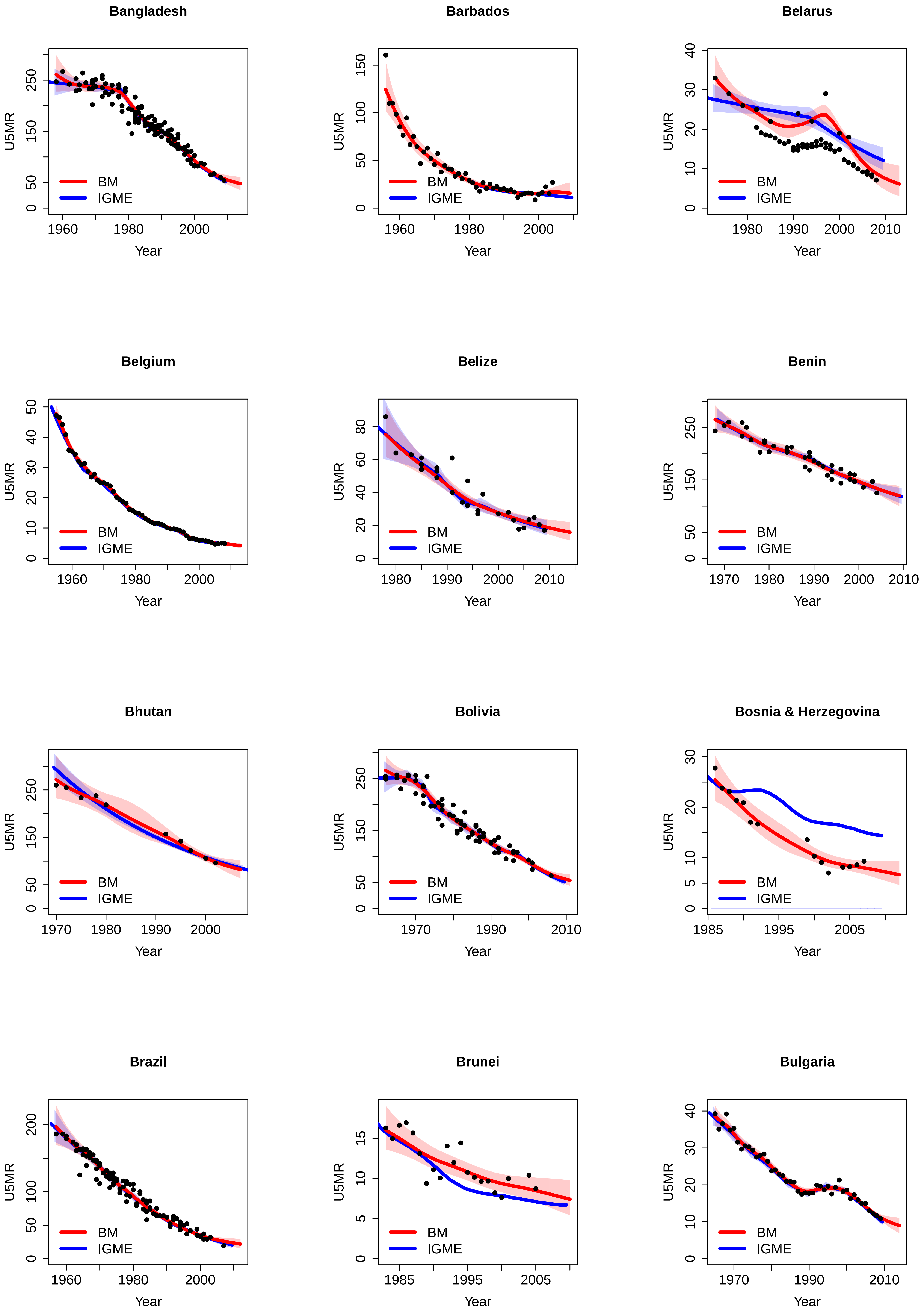
Bangladesh
Barbados
Belarus
Belgium
Belize
Benin
Bhutan
Bolivia
Bosnia & Herzegovina
Brazil
Brunei
Bulgaria
U5MR
Year
BM
IGME

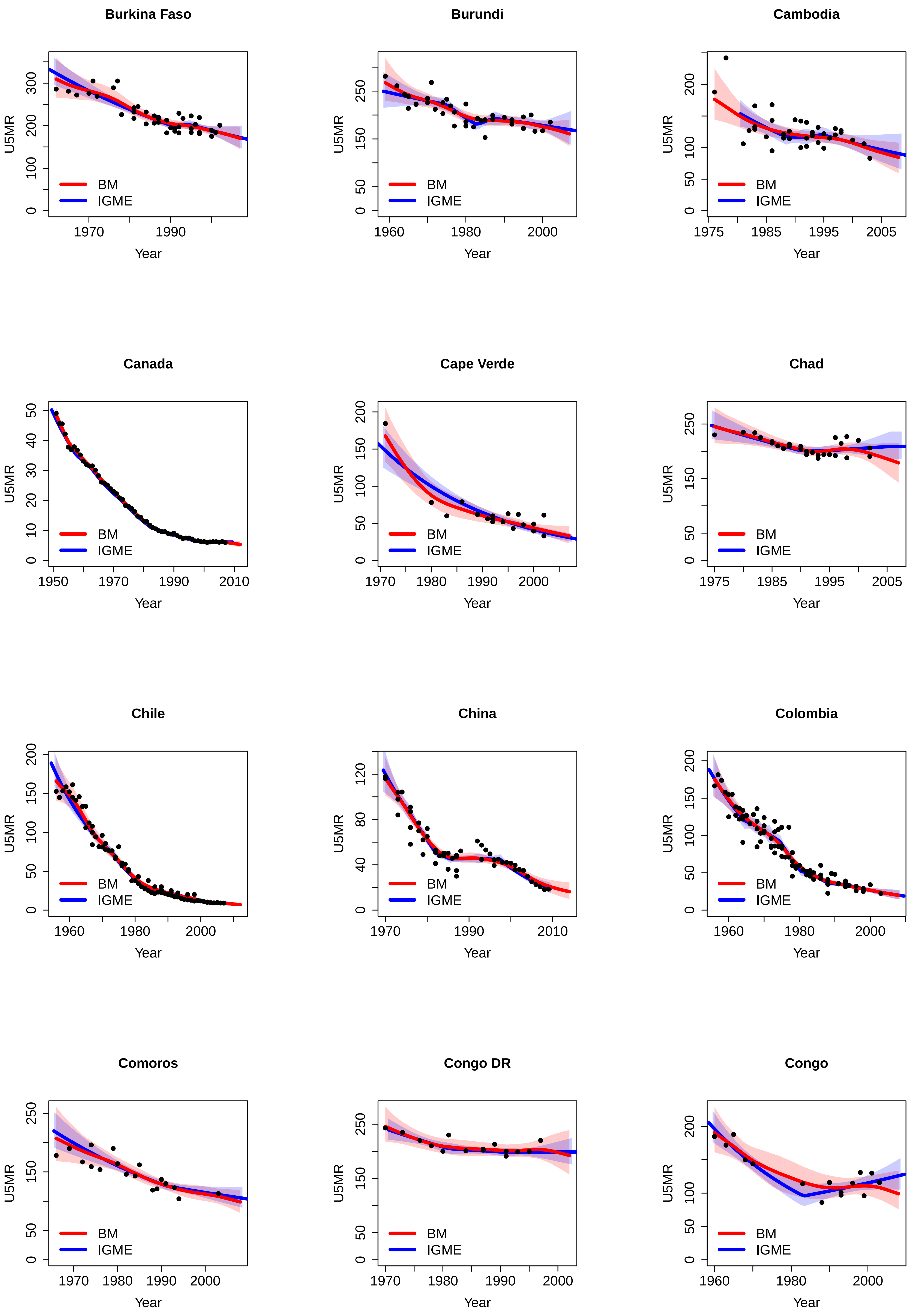

Burkina Faso
Burundi
Cambodia
Canada
Cape Verde
Chad
Chile
China
Colombia
Comoros
Congo DR
Congo
U5MR
Year
BM
IGME

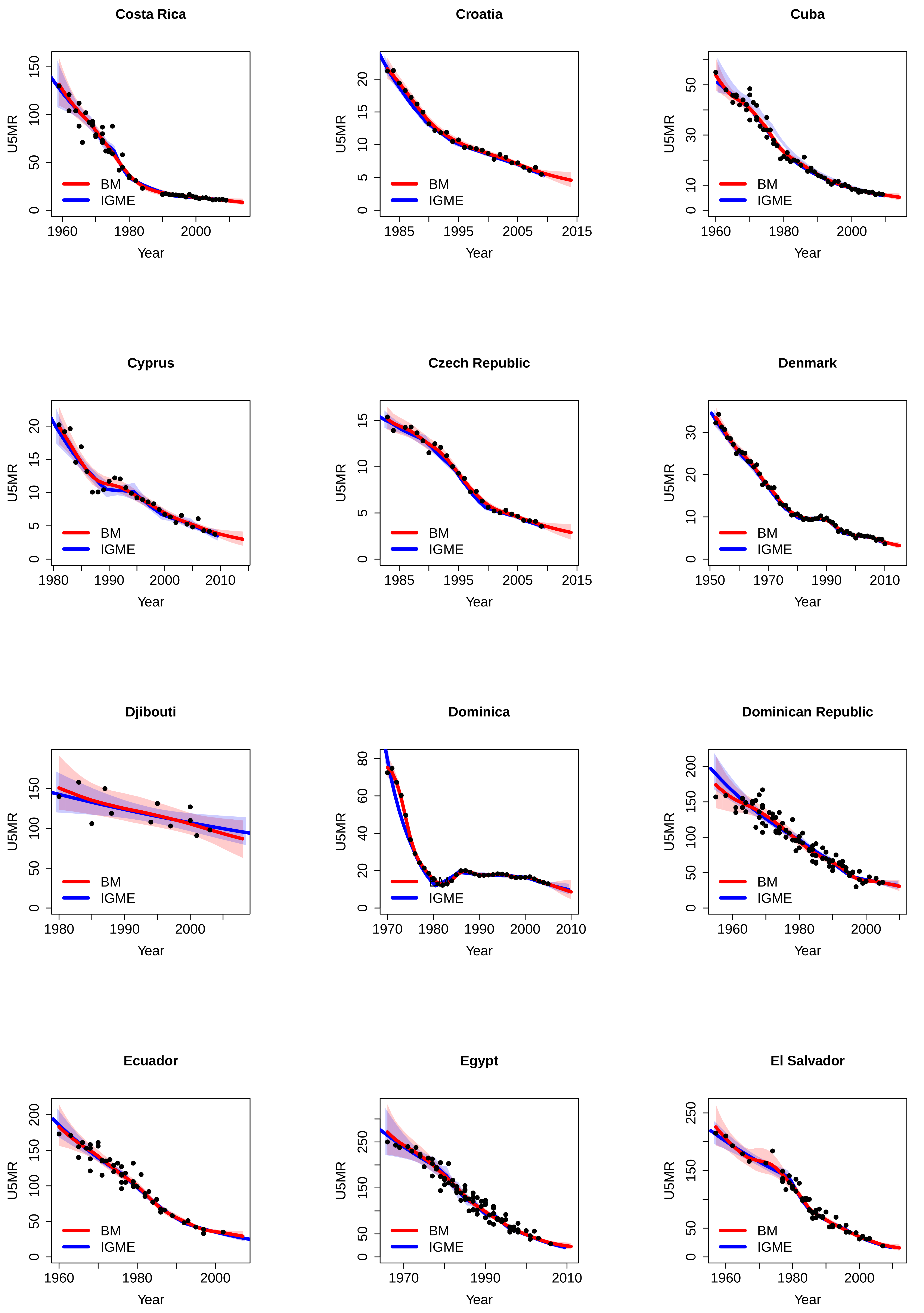
Costa Rica
Croatia
Cuba
Cyprus
Czech Republic
Denmark
Djibouti
Dominica
Dominican Republic
Ecuador
Egypt
El Salvador
U5MR
Year
BM
IGME

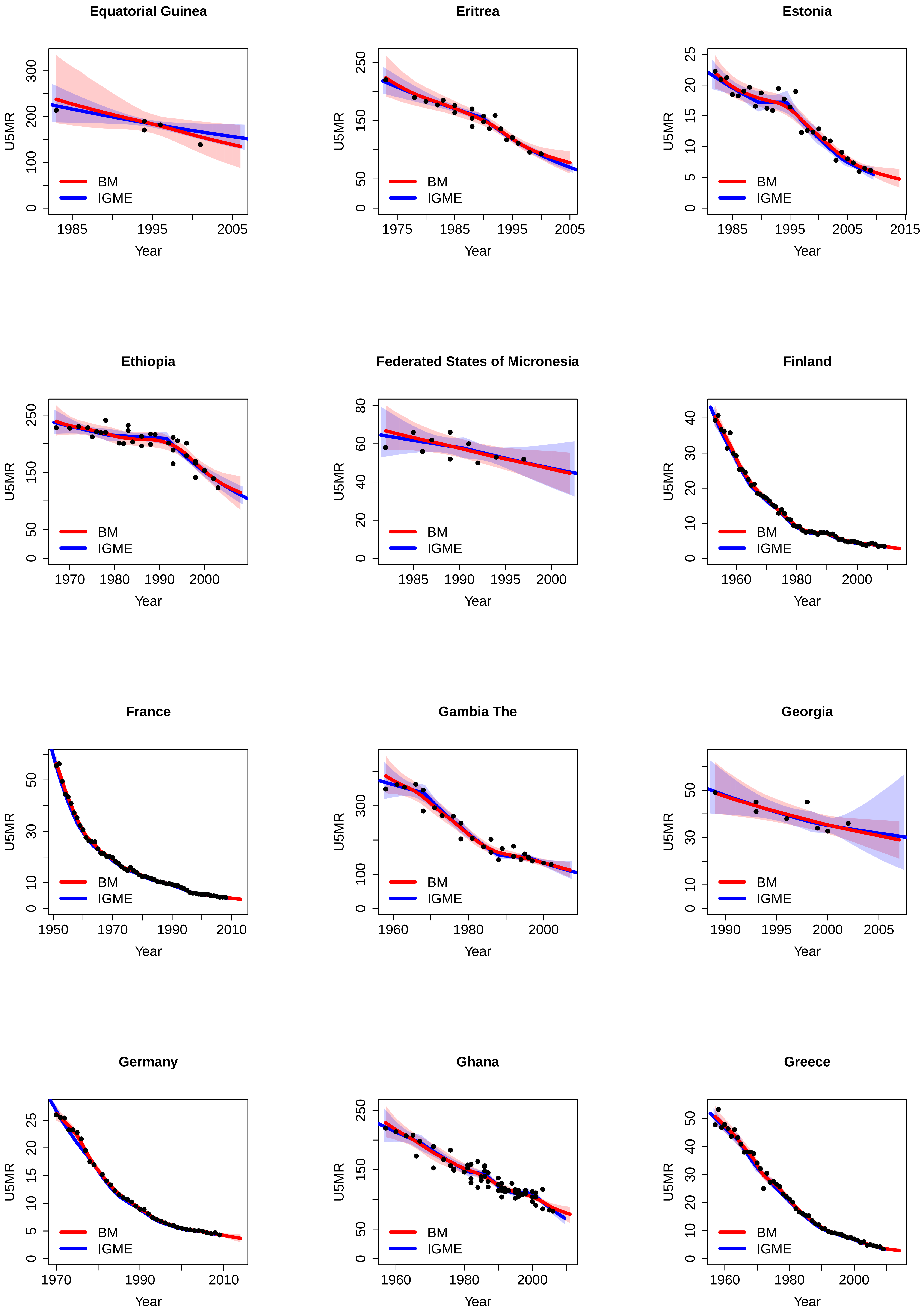
Equatorial Guinea
U5MR
0
100
200
300
1985
1995
2005
Year
BM
IGME
Eritrea
U5MR
0
50
150
250
1975
1985
1995
2005
Year
BM
IGME
Estonia
U5MR
0
5
10
15
20
25
1985
1995
2005
2015
Year
BM
IGME
Ethiopia
U5MR
0
50
150
250
1970
1980
1990
2000
Year
BM
IGME
Federated States of Micronesia
U5MR
0
20
40
60
80
1985
1990
1995
2000
Year
BM
IGME
Finland
U5MR
0
10
20
30
40
1960
1980
2000
Year
BM
IGME
France
U5MR
0
10
30
50
1950
1970
1990
2010
Year
BM
IGME
Gambia The
U5MR
0
100
300
1960
1980
2000
Year
BM
IGME
Georgia
U5MR
0
10
30
50
1990
1995
2000
2005
Year
BM
IGME
Germany
U5MR
0
5
10
15
20
25
1970
1990
2010
Year
BM
IGME
Ghana
U5MR
0
50
150
250
1960
1980
2000
Year
BM
IGME
Greece
U5MR
0
10
20
30
40
50
1960
1980
2000
Year
BM
IGME

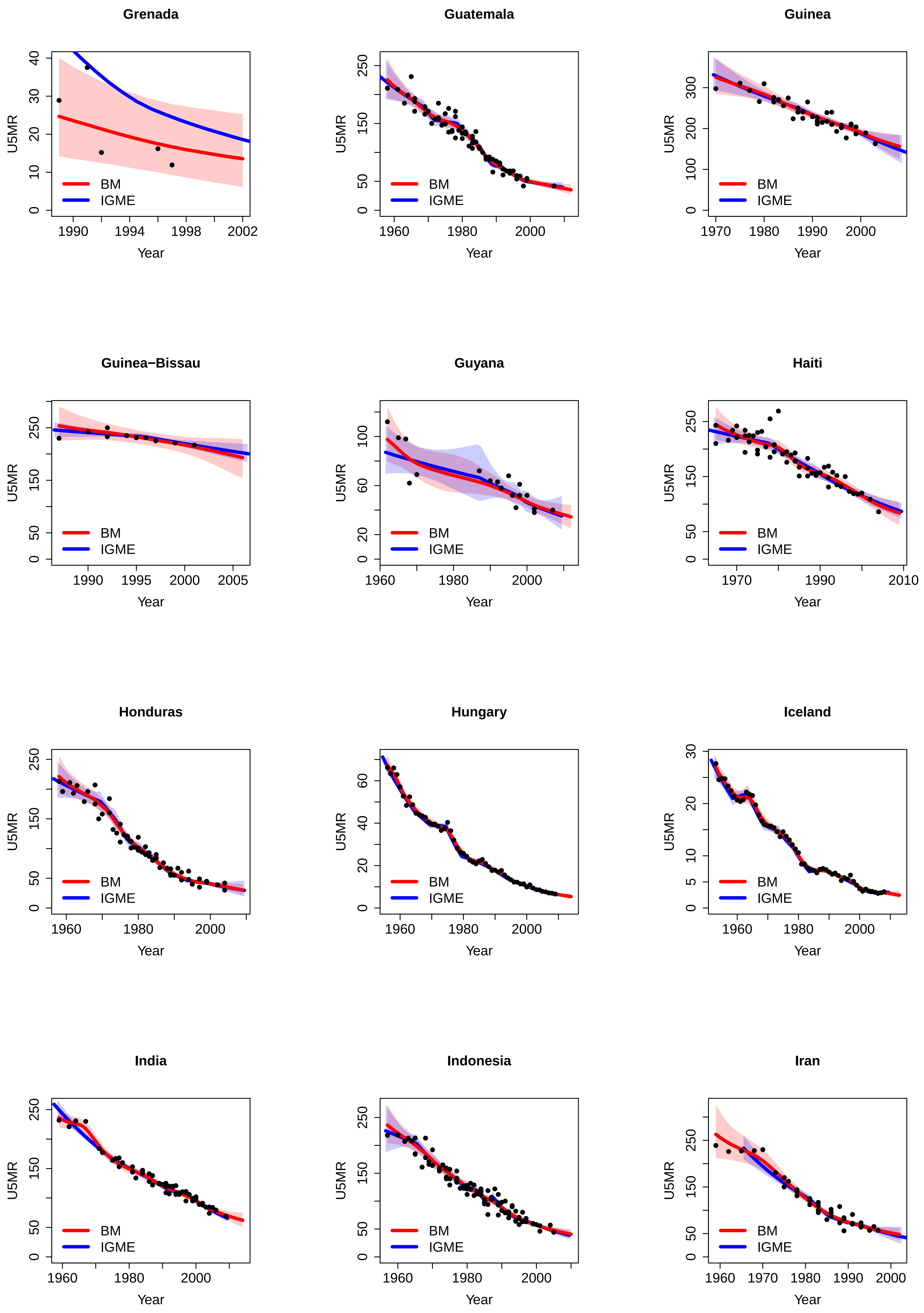
Grenada
Guatemala
Guinea
Guinea−Bissau
Guyana
Haiti
Honduras
Hungary
Iceland
India
Indonesia
Iran
U5MR
Year
BM
IGME

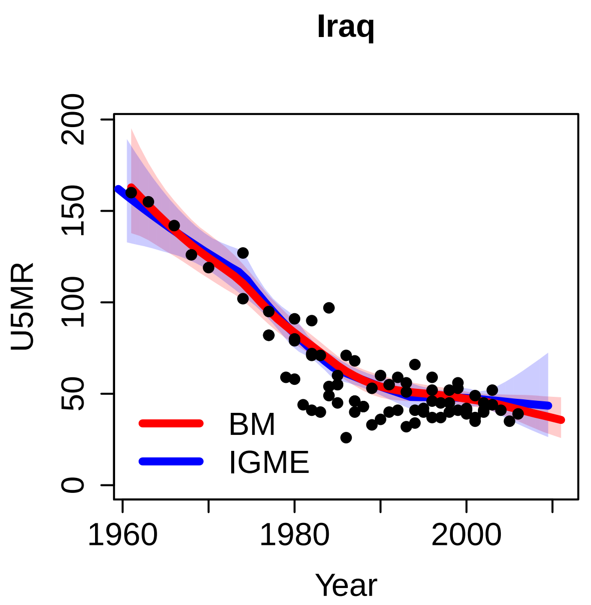
Iraq
U5MR
0
50
100
150
200
1960
1980
2000
Year
BM
IGME

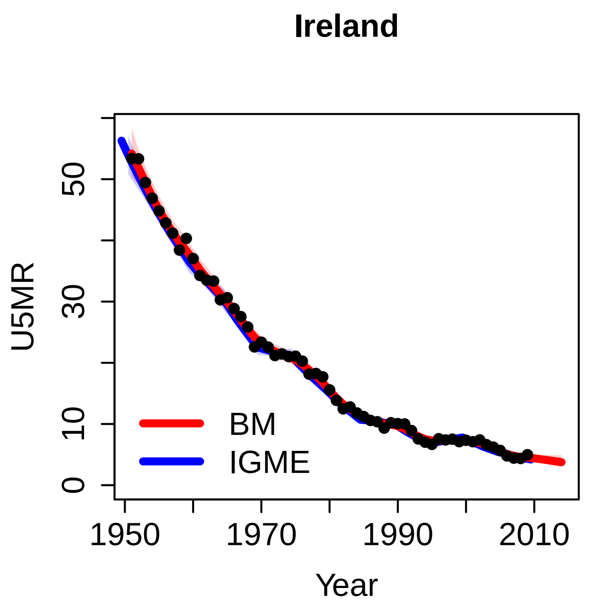
Ireland
U5MR
0
10
30
50
1950
1970
1990
2010
Year
BM
IGME

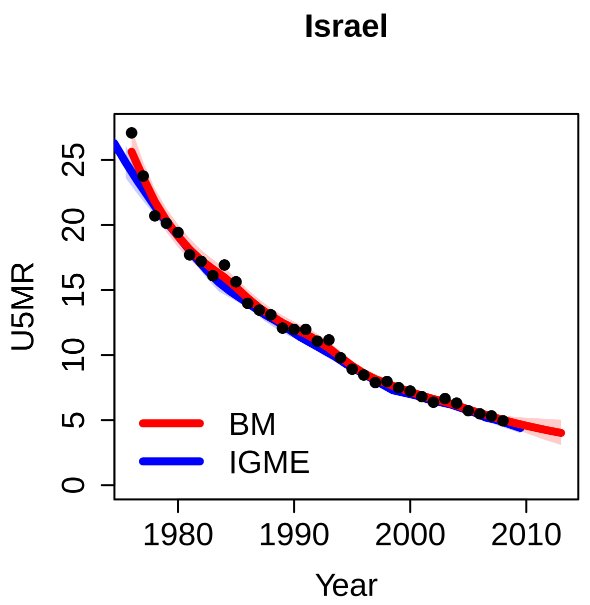
Israel
U5MR
0
5
10
15
20
25
1980
1990
2000
2010
Year
BM
IGME

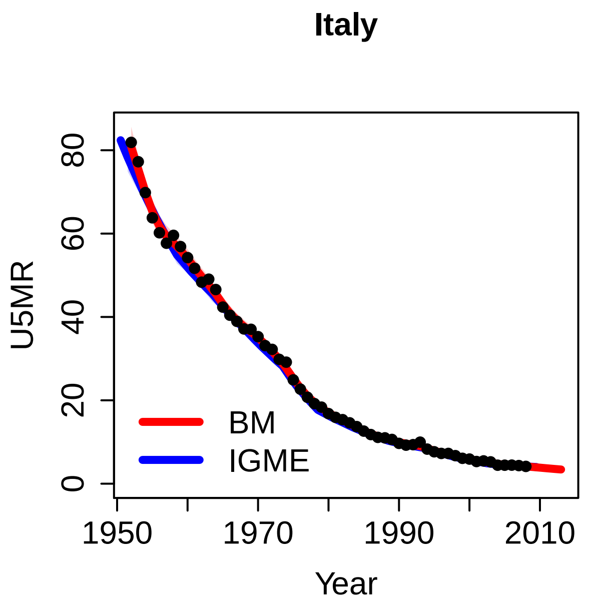
Italy
U5MR
0
20
40
60
80
1950
1970
1990
2010
Year
BM
IGME

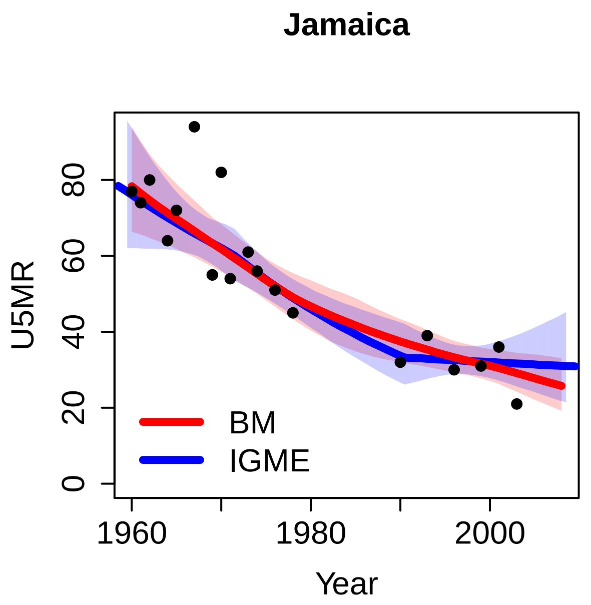
Jamaica
U5MR
0
20
40
60
80
1960
1980
2000
Year
BM
IGME

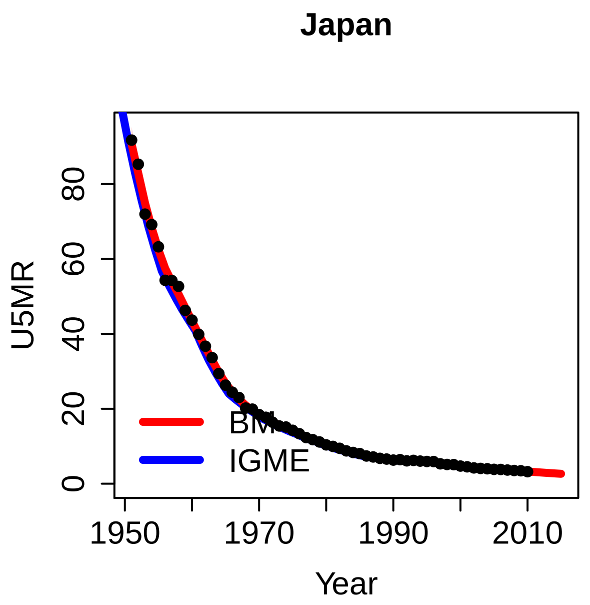
Japan
U5MR
0
20
40
60
80
1950
1970
1990
2010
Year
BM
IGME

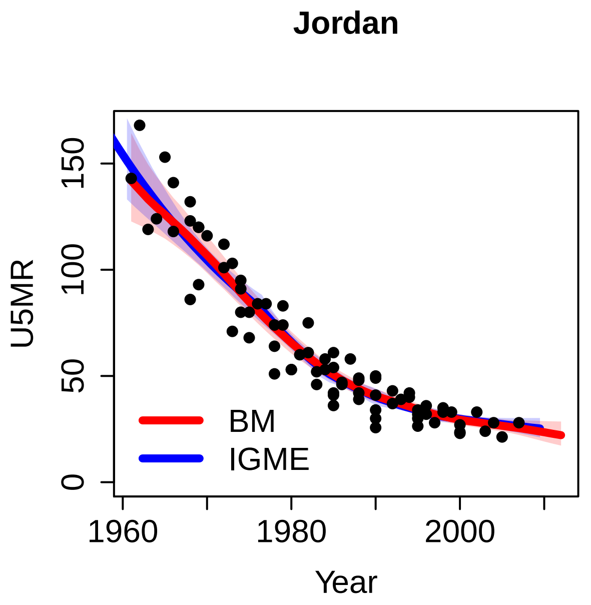
Jordan
U5MR
0
50
100
150
1960
1980
2000
Year
BM
IGME

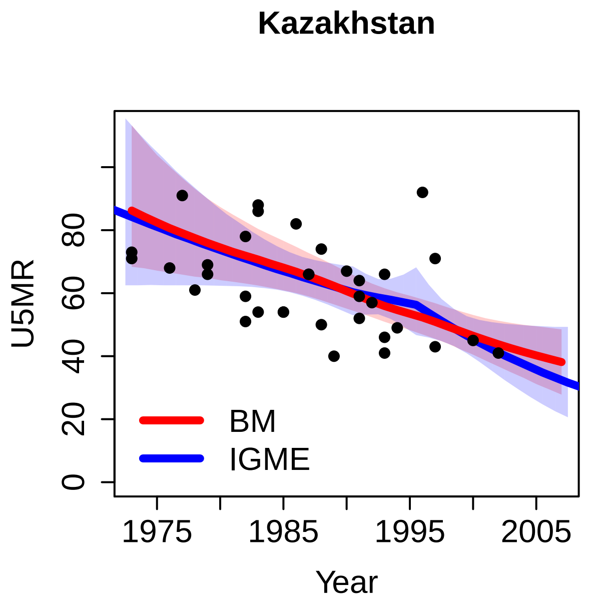
Kazakhstan
U5MR
0
20
40
60
80
1975
1985
1995
2005
Year
BM
IGME

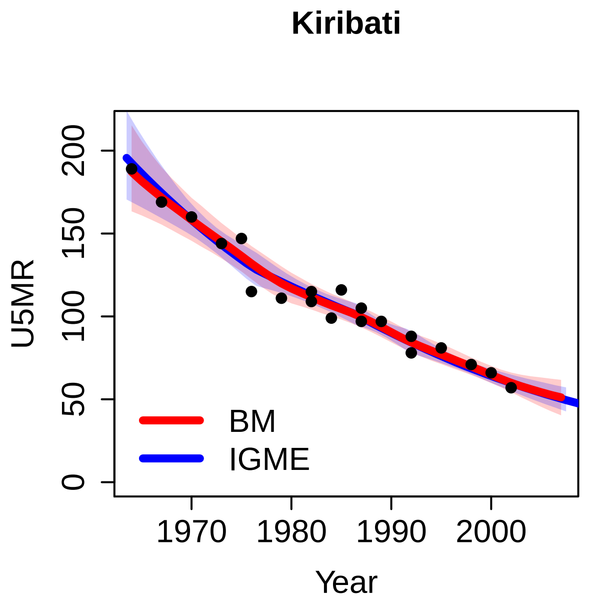
Kiribati
U5MR
0
50
100
150
200
1970
1980
1990
2000
Year
BM
IGME

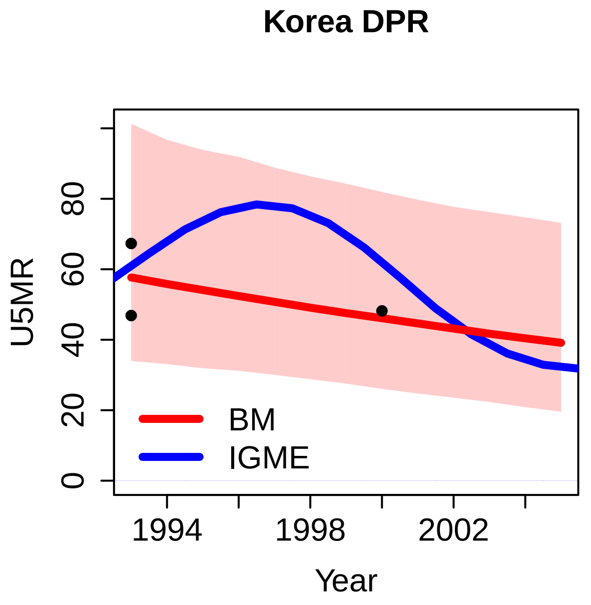
Korea DPR
U5MR
0
20
40
60
80
1994
1998
2002
Year
BM
IGME

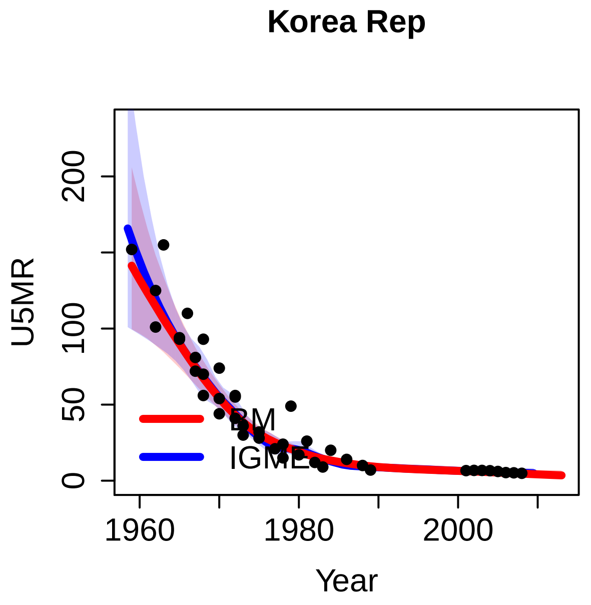
Korea Rep
U5MR
0
50
100
200
1960
1980
2000
Year
BM
IGME

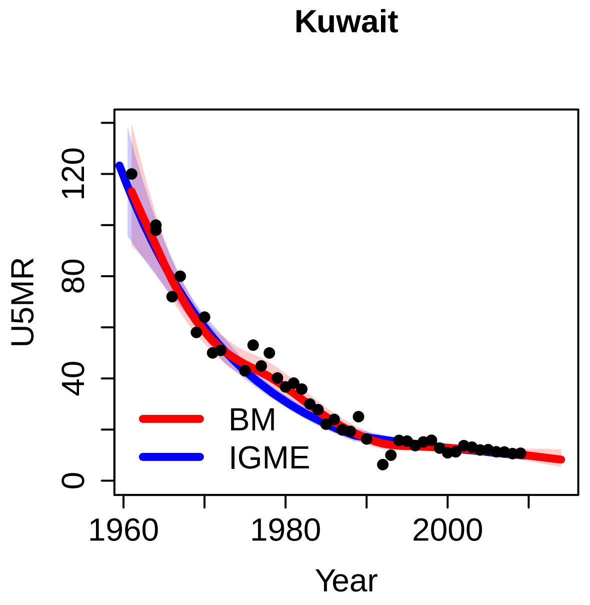
Kuwait
U5MR
0
40
80
120
1960
1980
2000
Year
BM
IGME

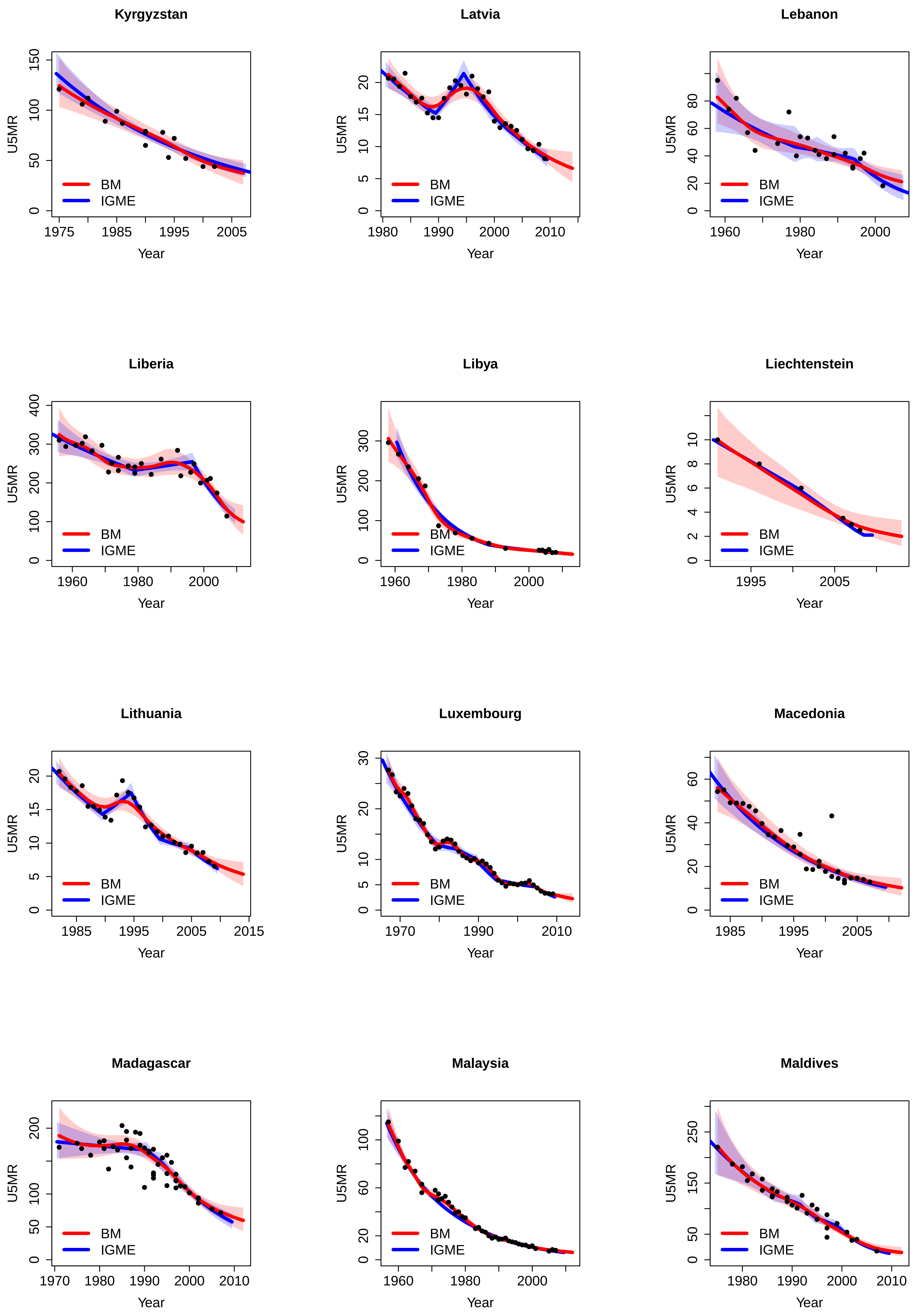
Kyrgyzstan
Latvia
Lebanon
Liberia
Libya
Liechtenstein
Lithuania
Luxembourg
Macedonia
Madagascar
Malaysia
Maldives
U5MR
Year
BM
IGME

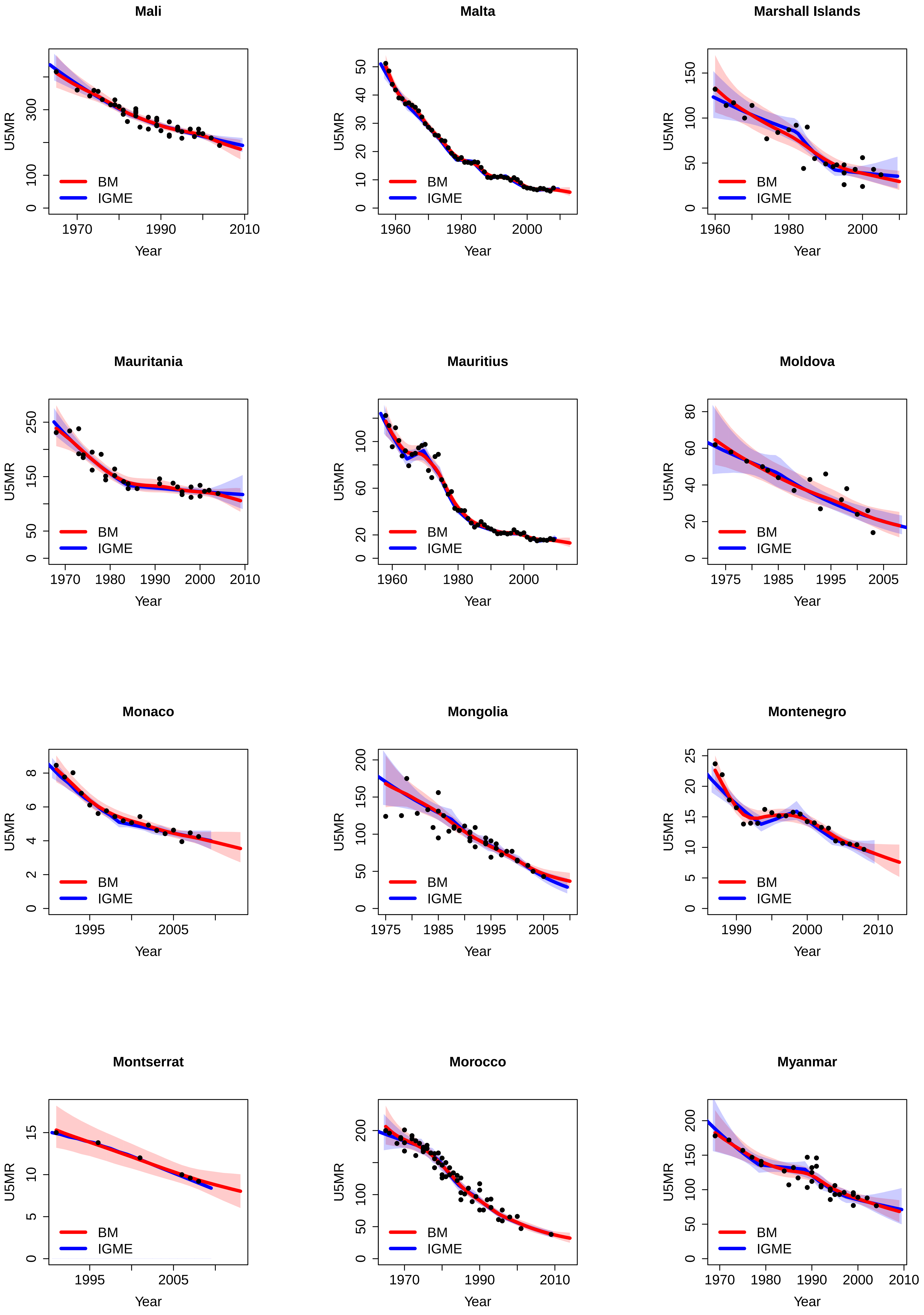
Mali
U5MR
Year
BM
IGME
Malta
U5MR
Year
BM
IGME
Marshall Islands
U5MR
Year
BM
IGME
Mauritania
U5MR
Year
BM
IGME
Mauritius
U5MR
Year
BM
IGME
Moldova
U5MR
Year
BM
IGME
Monaco
U5MR
Year
BM
IGME
Mongolia
U5MR
Year
BM
IGME
Montenegro
U5MR
Year
BM
IGME
Montserrat
U5MR
Year
BM
IGME
Morocco
U5MR
Year
BM
IGME
Myanmar
U5MR
Year
BM
IGME

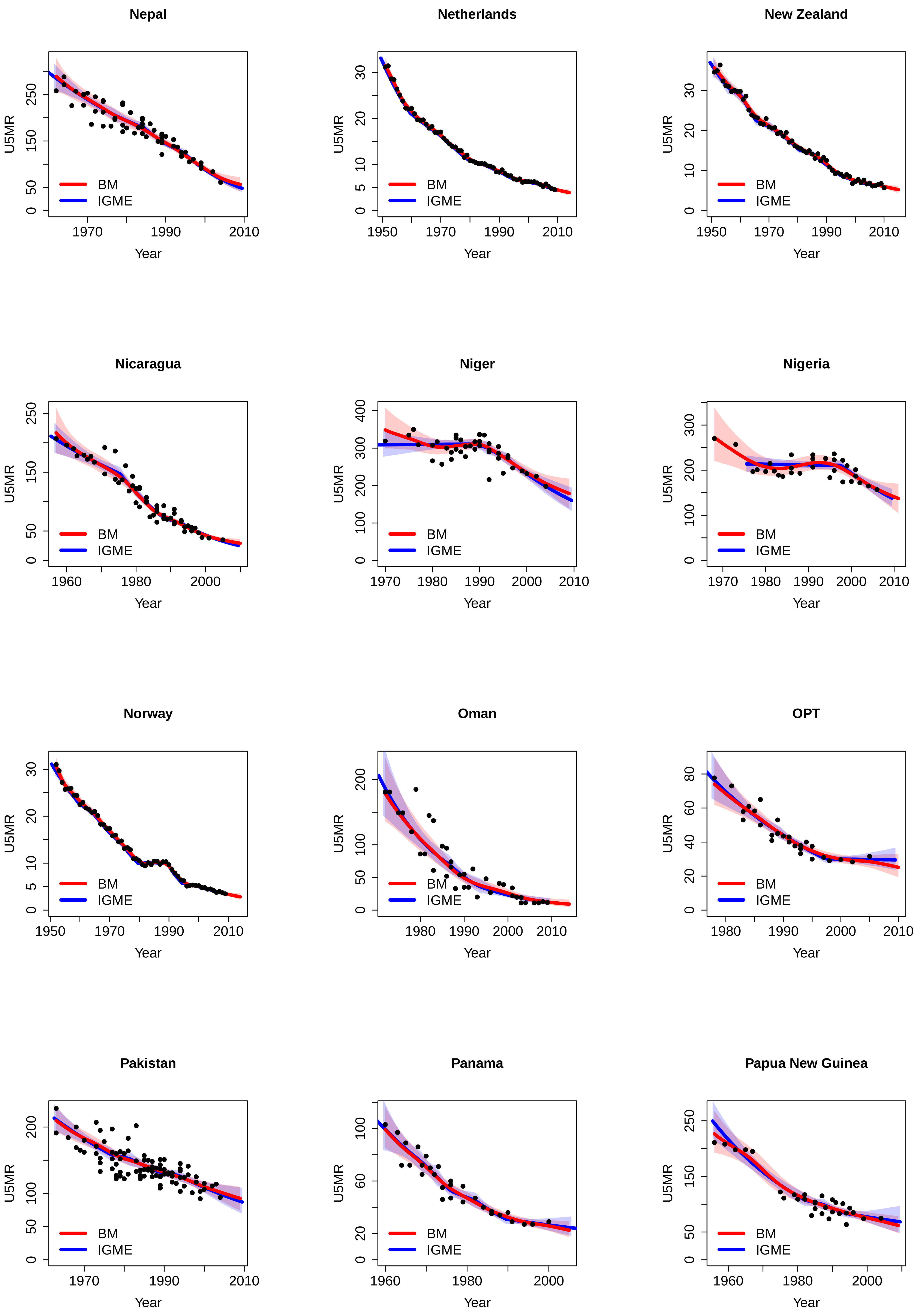
Nepal
Netherlands
New Zealand
Nicaragua
Niger
Nigeria
Norway
Oman
OPT
Pakistan
Panama
Papua New Guinea
U5MR
Year
BM
IGME

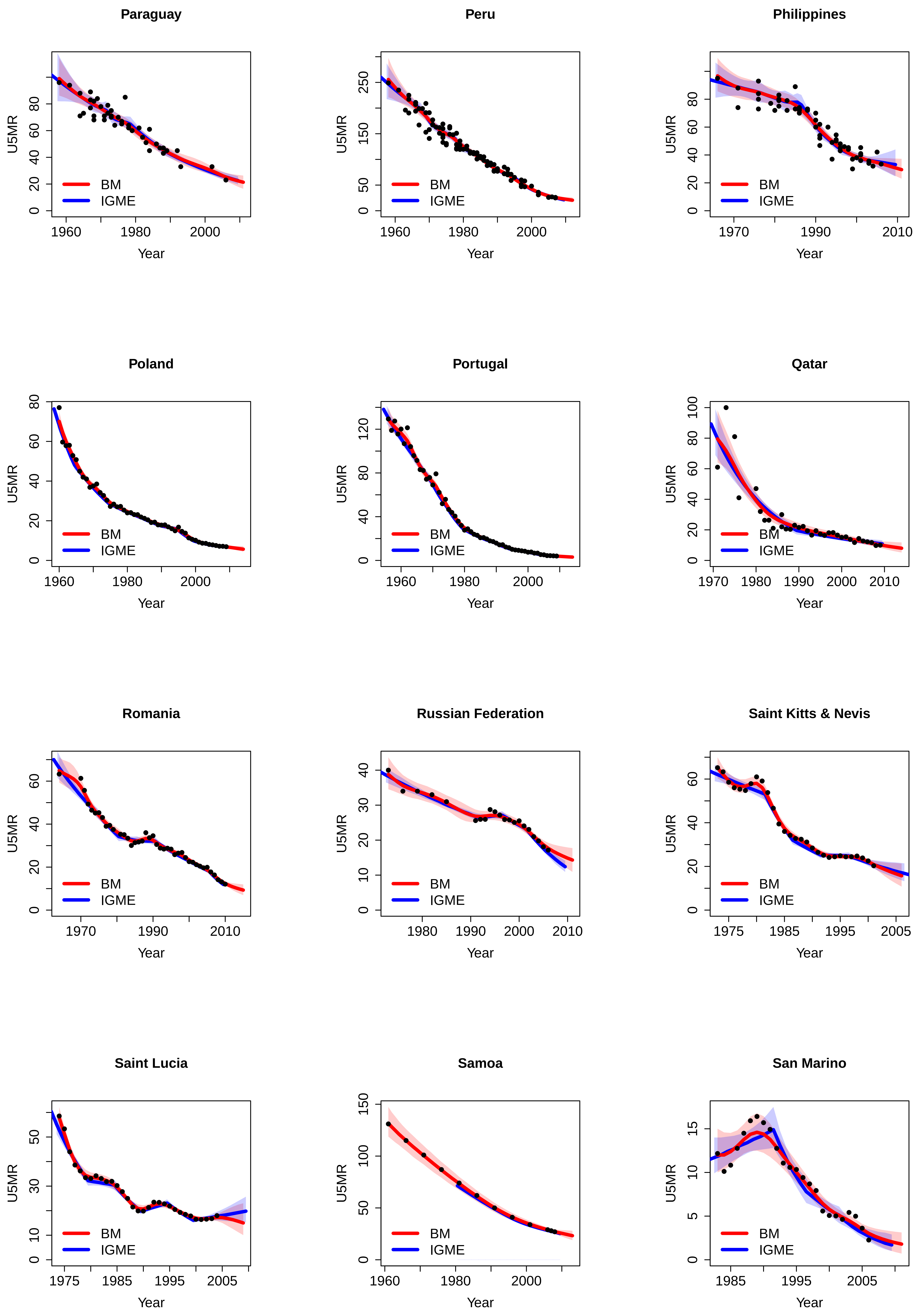
Paraguay
U5MR
0 20 40 60 80
1960 1980 2000
Year
BM
IGME
Peru
U5MR
0 50 150 250
1960 1980 2000
Year
BM
IGME
Philippines
U5MR
0 20 40 60 80
1970 1990 2010
Year
BM
IGME
Poland
U5MR
0 20 40 60 80
1960 1980 2000
Year
BM
IGME
Portugal
U5MR
0 40 80 120
1960 1980 2000
Year
BM
IGME
Qatar
U5MR
0 20 40 60 80 100
1970 1980 1990 2000 2010
Year
BM
IGME
Romania
U5MR
0 20 40 60
1970 1990 2010
Year
BM
IGME
Russian Federation
U5MR
0 10 20 30 40
1980 1990 2000 2010
Year
BM
IGME
Saint Kitts & Nevis
U5MR
0 20 40 60
1975 1985 1995 2005
Year
BM
IGME
Saint Lucia
U5MR
0 10 30 50
1975 1985 1995 2005
Year
BM
IGME
Samoa
U5MR
0 50 100 150
1960 1980 2000
Year
BM
IGME
San Marino
U5MR
0 5 10 15
1985 1995 2005
Year
BM
IGME

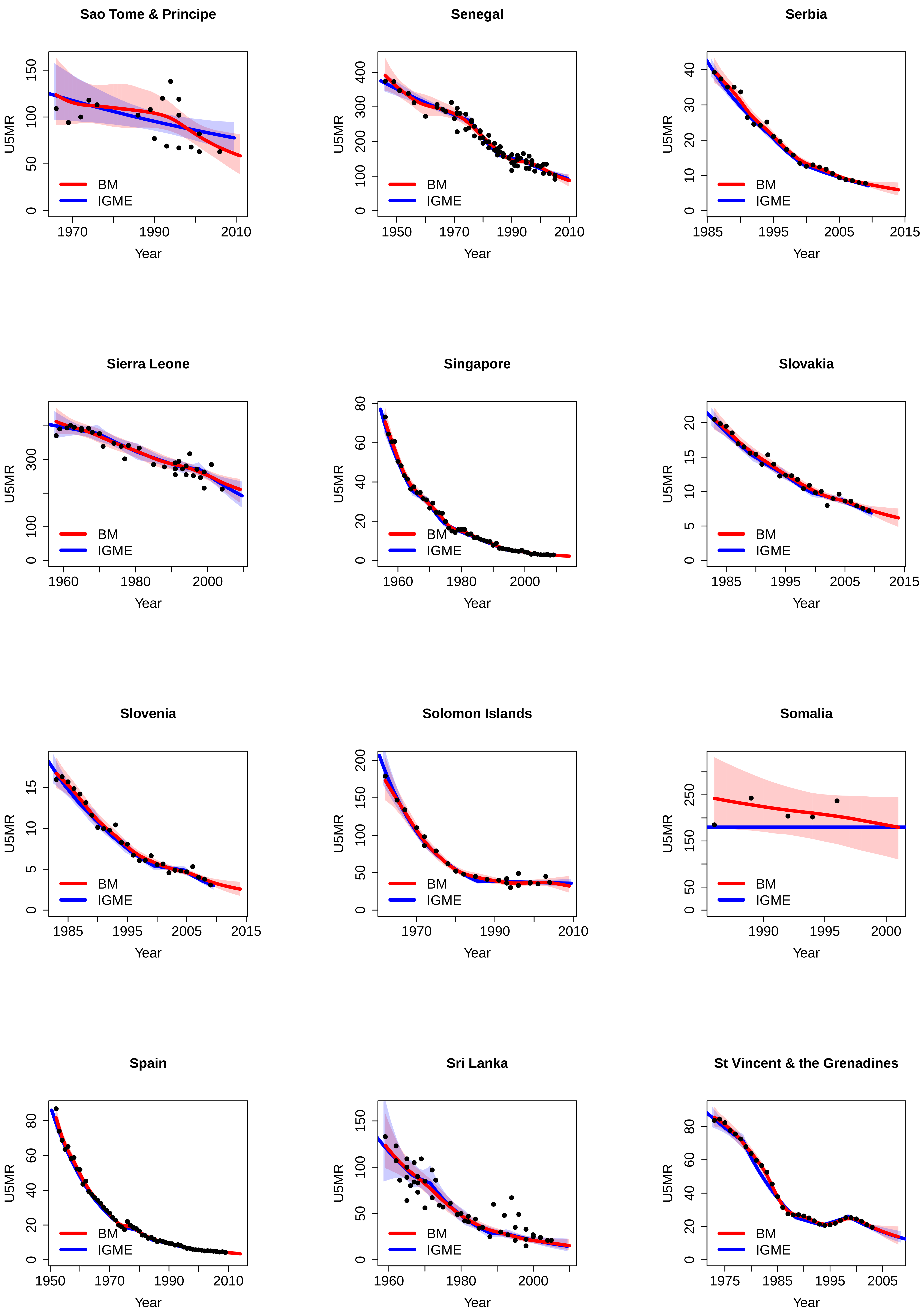
Sao Tome & Principe
Senegal
Serbia
Sierra Leone
Singapore
Slovakia
Slovenia
Solomon Islands
Somalia
Spain
Sri Lanka
St Vincent & the Grenadines
U5MR
Year
BM
IGME

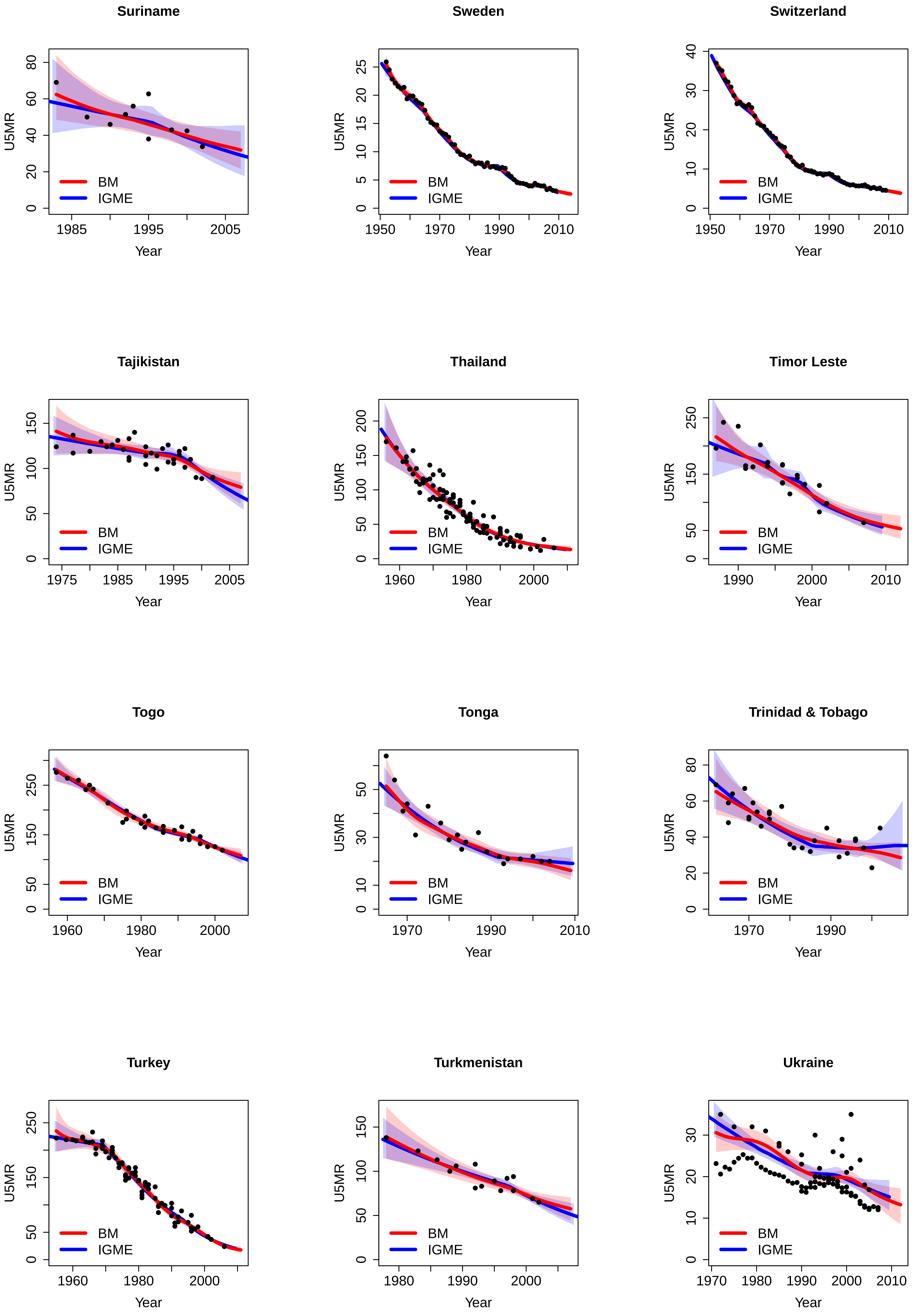

Suriname
Sweden
Switzerland
Tajikistan
Thailand
Timor Leste
Togo
Tonga
Trinidad & Tobago
Turkey
Turkmenistan
Ukraine
U5MR
Year
BM
IGME

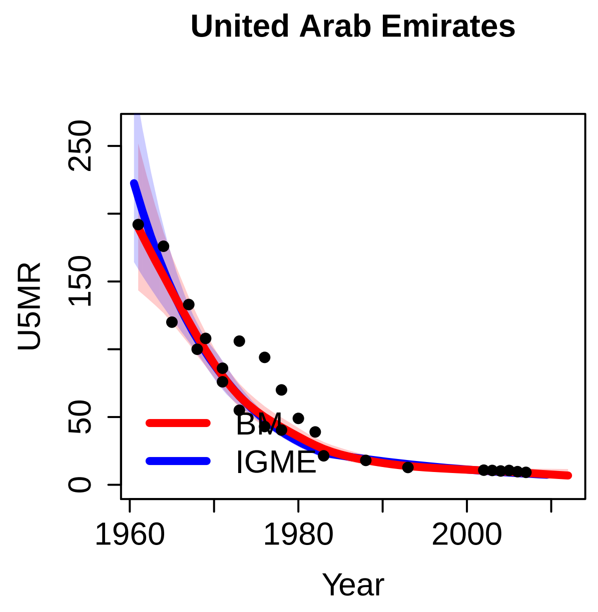
United Arab Emirates
U5MR
0
50
150
250
1960
1980
2000
Year
BM
IGME

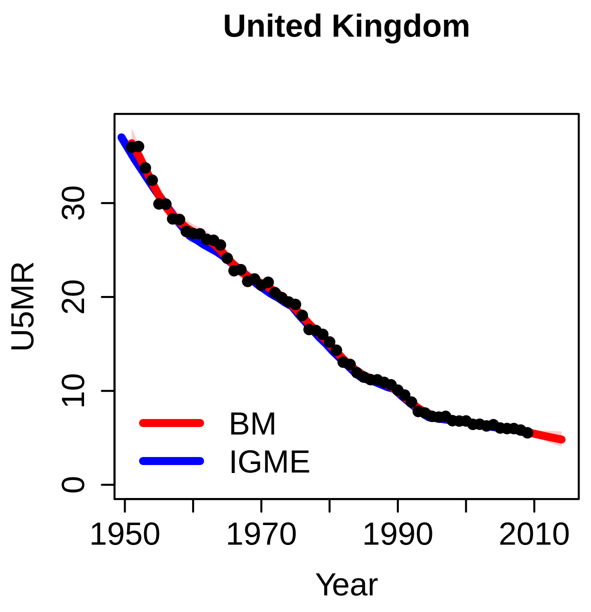
United Kingdom
U5MR
0
10
20
30
1950
1970
1990
2010
Year
BM
IGME

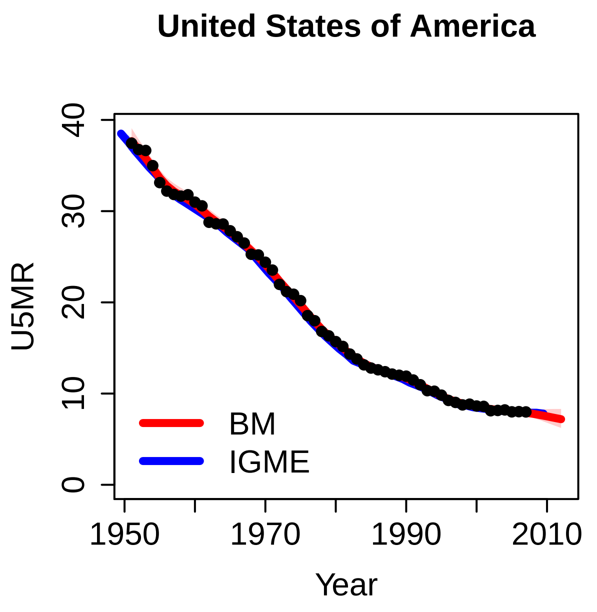
United States of America
U5MR
0
10
20
30
40
1950
1970
1990
2010
Year
BM
IGME

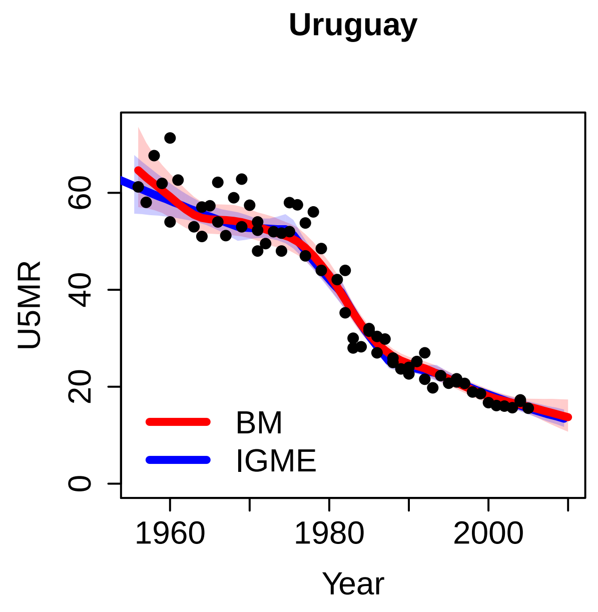
Uruguay
U5MR
0
20
40
60
1960
1980
2000
Year
BM
IGME

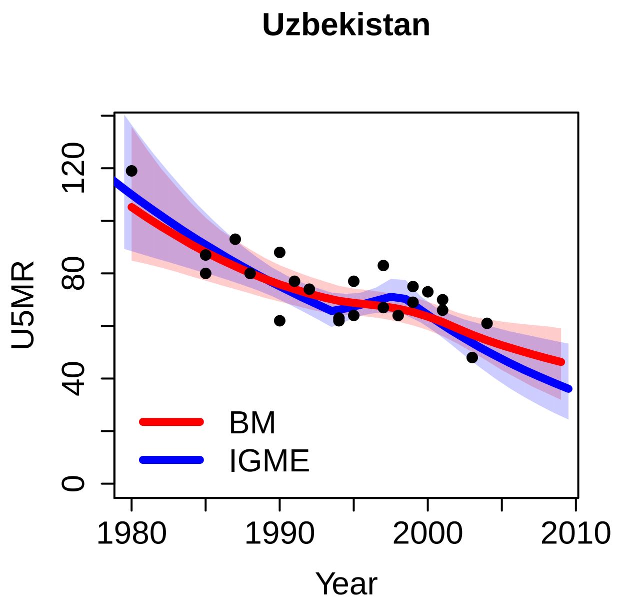
Uzbekistan
U5MR
0
40
80
120
1980
1990
2000
2010
Year
BM
IGME

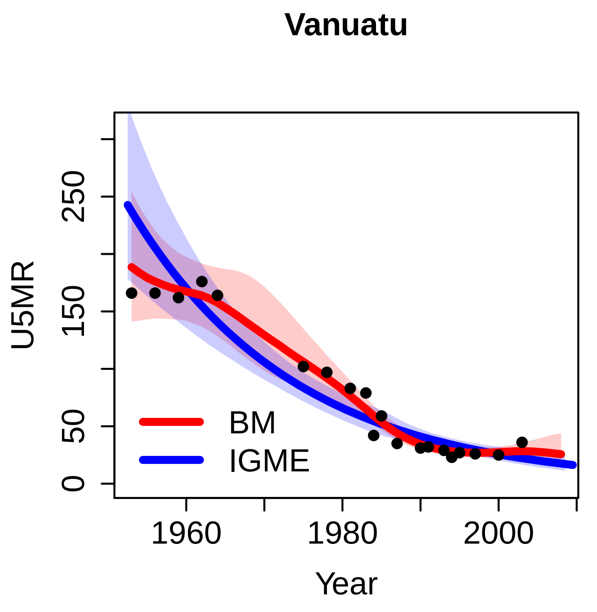
Vanuatu
U5MR
0
50
150
250
1960
1980
2000
Year
BM
IGME

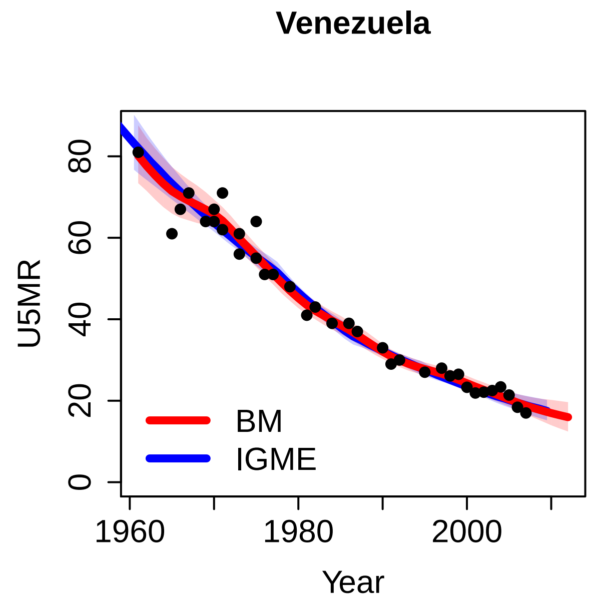
Venezuela
U5MR
0
20
40
60
80
1960
1980
2000
Year
BM
IGME

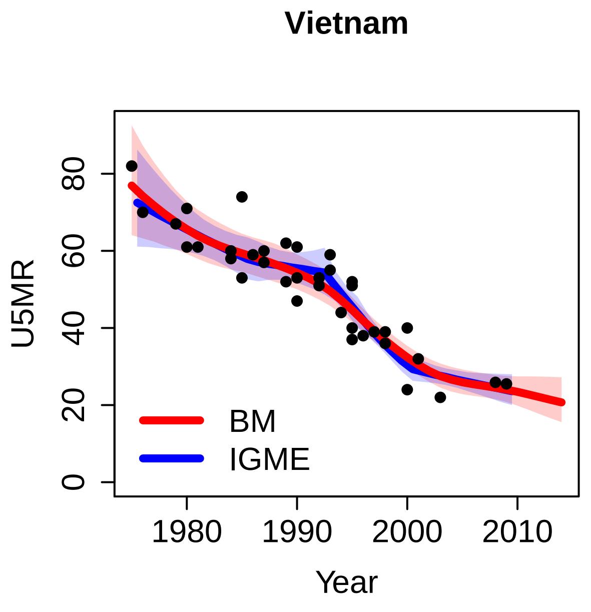
Vietnam
U5MR
0
20
40
60
80
1980
1990
2000
2010
Year
BM
IGME

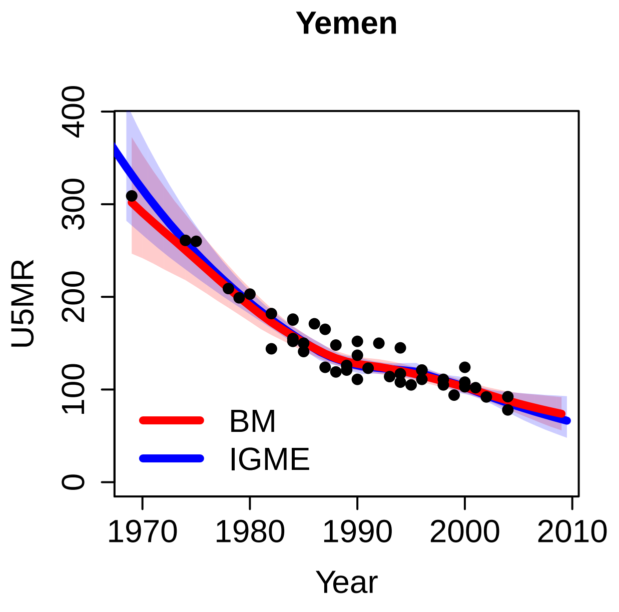
Yemen
U5MR
0
100
200
300
400
1970
1980
1990
2000
2010
Year
BM
IGME
